# Climate-aware hybrid Kolmogorov–Arnold networks for interpretable solar radiation forecasting

**DOI:** 10.1038/s41598-026-45578-y

**Published:** 2026-04-07

**Authors:** Mohammed Rahmath, Abdalla Alameen, Mohamed Sayed Abdellatif

**Affiliations:** 1 Department of Computer Engineering and Information, Prince Sattam Bin Abdulaziz University, College of Engineering in Wadi Adwassir, 11942 Al-Kharj, Saudi Arabia; 2 Department of Psychology, Prince Sattam Bin Abdulaziz University, College of Education in Al-Kharj, 11942 Al-Kharj, Saudi Arabia

**Keywords:** Solar radiation forecasting, Climate-aware modeling, Kolmogorov–Arnold Networks (KAN), Extreme Value Theory, Hybrid forecasting models, Interpretability, Global Horizontal Irradiance (GHI), Climate sciences, Environmental sciences

## Abstract

Accurate short-term solar radiation forecasting is essential for the reliable integration of photovoltaic systems into modern power grids, particularly in regions characterized by strong climatic heterogeneity. This study proposes a Climate-Aware Hybrid Kolmogorov–Arnold Network (CA-HKAN) framework for forecasting hourly Global Horizontal Irradiance (GHI) under diverse atmospheric regimes. The framework integrates an intrinsically interpretable spline-based Kolmogorov–Arnold Network with a feed-forward neural network through a deterministic switching mechanism governed by Extreme Value Theory (EVT). EVT is employed to derive climate-specific clearness-index thresholds, which are scaled to delineate stable and volatile irradiance regimes. These thresholds deterministically activate the interpretable spline-based component under physically stable conditions, while a neural network fallback is engaged during volatile or extreme atmospheric states. The proposed approach is evaluated using hourly meteorological and irradiance data from five climatically distinct regions in Saudi Arabia, representing desert, coastal, mountainous, and transitional environments. Experimental results demonstrate that the proposed CA-HKAN framework achieves predictive accuracy competitive with modern deep learning baselines, such as CNN–BiLSTM models, across all regions while maintaining physical consistency, including non-negativity and realistic irradiance bounds. Compared with standalone models, the hybrid approach offers a favorable balance between accuracy, robustness, and transparency. Ablation analyses further confirm the complementary roles of the hybrid components and the effectiveness of EVT-based regime control. Overall, the CA-HKAN framework provides a practical and interpretable solution for climate-aware solar radiation forecasting, supporting trustworthy deployment in sustainable energy systems operating under heterogeneous and non-stationary atmospheric conditions.

## Introduction

### Motivation

Accurate short-term solar radiation forecasting is a cornerstone of sustainable energy system operation, directly influencing photovoltaic (PV) integration, grid stability, reserve scheduling, and energy market decisions^1,2^. These requirements are particularly critical in regions characterized by strong climatic heterogeneity—such as desert, coastal, mountainous, and transitional environments—where solar irradiance exhibits pronounced non-stationarity driven by rapid atmospheric changes, aerosol variability, and cloud dynamics^3,4^. Such variability motivates the development of forecasting frameworks that are not only accurate, but also robust, physically consistent, and suitable for operational deployment^5^.

Machine and Deep Learning for Climatic Event Forecasting Beyond solar irradiance prediction, machine and deep learning techniques have been widely applied to a broad range of climate-related forecasting tasks, including air quality modeling, environmental pollution prediction, renewable energy demand forecasting, and environmental risk assessment. Recent studies demonstrate that hybrid data-driven frameworks can effectively capture complex atmospheric dynamics and extreme-event behavior, significantly improving prediction stability under highly variable environmental conditions^6,7^.

For instance, ensemble machine learning approaches have shown strong capability in predicting atmospheric pollutant concentrations and assessing their health impacts^8^, while deep learning architectures have demonstrated strong performance in short-term wind speed forecasting for renewable energy applications^9^. Hybrid statistical methods combining signal decomposition with neural networks have further improved prediction accuracy for climatic time-series forecasting^10^.

Artificial intelligence models have also been integrated with extreme value theory for environmental risk modeling and extreme-event prediction, highlighting the growing importance of interpretable AI frameworks in climate and energy forecasting^11,12^. These developments reflect a broader paradigm shift toward interpretable, physically grounded forecasting systems capable of modeling complex nonlinear environmental processes^13^.

However, solar irradiance forecasting presents unique challenges not fully encountered in other climatic prediction tasks, including strong diurnal periodicity, strict physical bounds, and rapid irradiance ramp events caused by cloud dynamics. These characteristics necessitate forecasting architectures that explicitly integrate physical constraints, regime-dependent dynamics, and interpretable functional representations.

### Challenges and limitations of existing approaches

A fundamental challenge in solar forecasting is the climate dependence of model performance. Models trained in one atmospheric regime often fail to generalize across regions with different irradiance dynamics, particularly in arid and semi-arid climates that exhibit sharp transitions, high aerosol loads, and strong seasonal variability^5,14^. As a result, generic forecasting models frequently underperform in solar-rich regions where reliability is most critical.

To address non-stationarity, recent approaches increasingly adopt hybrid modeling strategies, combining neural networks, statistical models, or signal-processing techniques^15,16^. While such hybrids improve robustness relative to single-model approaches, their blending mechanisms are typically heuristic or probabilistic, lack physical grounding, and do not explicitly account for climate-specific stability or volatility. This limits their transparency and predictability in operational energy systems^6,17^.

Extreme Value Theory (EVT) has been widely used to analyze irradiance extremes, ramp events, and renewable energy risks^18,19^. However, existing applications primarily employ EVT for descriptive or post-hoc probabilistic analysis, rather than as an active control mechanism within forecasting architectures. Consequently, EVT’s potential to guide adaptive model behavior under varying atmospheric regimes remains largely unexplored^7,11^.

At the same time, deep learning models such as CNN–LSTM hybrids and Transformer-based architectures dominate current forecasting benchmarks^20,21^. Despite their high predictive accuracy, these models are computationally intensive, opaque, and difficult to validate under extreme or transitional conditions, particularly in multi-climate settings^21,22^. Moreover, many data-driven approaches do not explicitly enforce physical consistency, such as non-negativity or realistic irradiance bounds, which can lead to operationally unsafe predictions under rare or volatile conditions^23^.

Post-hoc explainable artificial intelligence (XAI) techniques, including SHAP and LIME, have been introduced to improve transparency^24,25^. However, these methods do not influence the underlying model structure and may produce unstable explanations under distribution shifts, limiting their effectiveness for operational trust^26^.

Recently, intrinsically interpretable architectures such as Kolmogorov–Arnold Networks (KANs) have attracted attention due to their spline-based functional representations^27,28^. While KANs offer built-in interpretability, they inherently assume smooth and stable input–output relationships, making them vulnerable under highly volatile atmospheric regimes^29^. To date, KAN-based models have not been integrated into climate-aware frameworks that selectively restrict their use to physically stable irradiance conditions^29^.

### Research gap

The above challenges reveal a clear gap in the literature: the absence of a unified solar forecasting framework that simultaneously incorporates climate-aware regime detection, extreme-value-driven decision rules, intrinsic interpretability, and physical consistency. Existing hybrid models lack physically grounded switching mechanisms, EVT has not been leveraged as an architectural control tool, and interpretable models remain insufficiently robust under non-stationary atmospheric conditions. This gap is particularly consequential for sustainable energy systems that require both high predictive accuracy and transparent, trustworthy behavior across diverse climates^25,27^.

### Study objective

The primary objective of this study is to develop a climate-aware and interpretable solar radiation forecasting framework that adaptively balances model transparency and robustness across heterogeneous atmospheric regimes. Specifically, the study aims to exploit the physical relevance of extreme-value behavior to guide deterministic model selection, while preserving interpretability and enforcing physical consistency for operational energy applications.

### Contributions

To achieve this objective, this study makes the following contributions:

- **Climate-aware hybrid forecasting framework:** A Climate-Aware Hybrid Kolmogorov–Arnold Network (CA-HKAN) is proposed, integrating an interpretable spline-based KAN with a neural network fallback to handle regime-dependent atmospheric variability.
- **EVT-driven deterministic control mechanism:** EVT-derived clearness-index thresholds are used to deterministically govern model selection, transforming EVT from a descriptive analysis tool into an architectural control mechanism.
- **Intrinsic interpretability with robustness:** The framework preserves intrinsic interpretability through KAN spline representations while ensuring robustness under volatile conditions via neural fallback modeling.
- **Physically consistent, deployment-oriented design:** Physical validity constraints are enforced to ensure realistic irradiance predictions, supporting safe and reliable use in real-world energy systems.
- **Multi-climate validation framework:** The proposed approach is evaluated across multiple climatically distinct regions using systematic ablation studies and comparisons with modern deep learning baselines.

Positioning Relative to Existing Hybrid Models Hybrid forecasting architectures combining interpretable and black-box components have been explored in prior studies, particularly within regime-switching and ensemble learning frameworks. However, existing approaches typically rely on probabilistic blending, heuristic weighting, or purely statistical switching criteria without explicit physical grounding.

The proposed framework differs fundamentally in three aspects. First, regime detection is derived from Extreme Value Theory (EVT), providing statistically justified boundaries based on tail behavior of clearness-index distributions rather than empirical thresholds. Second, the switching mechanism is deterministic and climate-aware, enabling explicit separation between stable atmospheric regimes-where interpretable functional modeling is appropriate-and highly volatile regimes requiring flexible nonlinear approximation. Third, the Kolmogorov–Arnold Network introduces intrinsic interpretability through spline-based functional decomposition, which is not present in conventional neural network hybrids.

Regarding model suitability, the neural network module is not intended to model long-term temporal dependencies but rather to handle short-term nonlinear residual dynamics under unstable atmospheric conditions. This design complements the interpretable KAN module rather than serving as a standalone time-series forecasting architecture.

The remainder of this paper is organized as follows. Section Methodology details the proposed CA-HKAN framework, including data preprocessing, EVT-based threshold derivation, model architectures, and the hybrid switching mechanism. Section Results presents the experimental evaluation and ablation analyses across multiple climatic regions. Section Discussion discusses the implications, limitations, and practical relevance of the proposed approach for sustainable energy systems. Finally, Section Conclusion concludes the paper and outlines directions for future research.

## Methodology

This section presents the methodological framework for the Climate-Aware Hybrid Kolmogorov–Arnold Network (CA-HKAN) model. The framework integrates an enhanced Kolmogorov–Arnold Network (KAN) with a feed-forward neural network (NN) through a deterministic climate-aware switching mechanism derived from Extreme Value Theory (EVT).

**Fig. 1:**
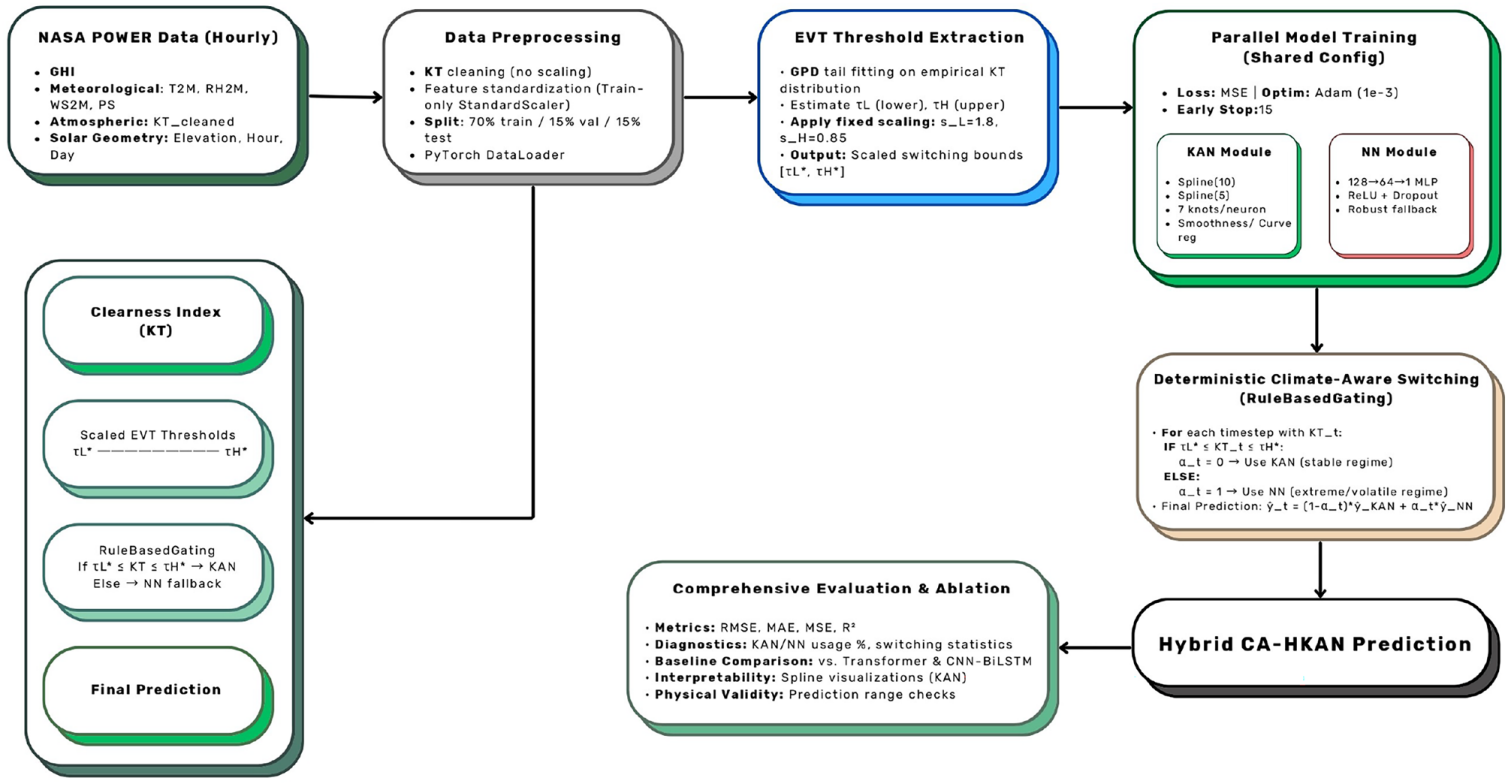
Overall methodology of the Climate-Aware Hybrid Kolmogorov–Arnold Network (CA-HKAN). The pipeline includes data preprocessing, EVT-based threshold extraction, model training for the KAN and neural network modules, deterministic hybrid switching, and ablation testing.

The complete pipeline, illustrated in Fig. 1, includes preprocessing, threshold extraction, model training, hybrid blending, and ablation testing.

### Data sources and preprocessing

Hourly Global Horizontal Irradiance (GHI) and meteorological variables were obtained from the NASA POWER database for five Saudi Arabian climate regions^30^. The predictor set includes:

- **Meteorological variables:** temperature (T2M), relative humidity (RH2M), wind speed (WS2M), and surface pressure (PS)^31,32^.
- **Atmospheric clarity measure:** cleaned clearness index (ALLSKY_KT_cleaned)^14,32^.
- **Solar geometry variables:** solar elevation angle, hour of day, and day of year^33,34^.

The preprocessing workflow is defined as follows:

- KT values are cleaned and used directly for EVT analysis^7,18^.
- All predictors except KT are standardized using a train-only StandardScaler^6,35^.
- The dataset is split into 70% training, 15% validation, and 15% testing^1,31^.
- PyTorch DataLoader objects manage batching, shuffling, and device allocation^36^.

This procedure ensures consistent training behavior across all models.

Forecasting Setup and Temporal Evaluation The forecasting task was formulated as a one-step-ahead hourly prediction problem; full details of temporal splitting, leakage prevention, and rolling-window validation are provided in Section Forecasting horizon and leakage prevention.

### EVT-based clearness-index thresholding

To identify atmospheric regimes where interpretable spline modeling is reliable, thresholds were derived from the empirical distribution of the clearness index $K_T$ using Extreme Value Theory (EVT)^18,19^. The clearness index, defined as the ratio between surface global horizontal irradiance and extraterrestrial irradiance, provides a normalized measure of atmospheric transmissivity and is widely used to characterize solar variability^14^.

A peaks-over-threshold (POT) framework was employed to model extreme deviations in $K_T$. For a random variable *X* representing the clearness index, exceedances over a sufficiently high threshold *u* follow the generalized Pareto distribution (GPD):

1 $$\begin{aligned} P(X-u \le y \mid X> u) = 1 - \left( 1 + \frac{\xi y}{\sigma }\right) ^{-1/\xi }, \end{aligned}$$

where $\xi$ is the shape parameter and $\sigma> 0$ is the scale parameter^7,11^. Negative values of $\xi$ correspond to bounded tail behavior, consistent with the physical limits of the clearness index.

Lower- and upper-tail thresholds were estimated independently for each city. Candidate threshold ranges were first identified using mean residual life (MRL) plots, where approximate linearity indicates validity of the GPD assumption. Parameter stability plots were then constructed by fitting GPD models across candidate thresholds; stable estimates of $\xi$ and $\sigma$ indicate suitable threshold regions. Final thresholds $\tau _L$ and $\tau _H$ were selected as the lowest values within these stable regions to maximize sample size while maintaining distributional validity.

Goodness-of-fit was assessed using quantile–quantile plots, probability–probability plots, and Kolmogorov–Smirnov tests to verify consistency between empirical exceedances and the fitted GPD model. Because clearness-index distributions differ across climatic regimes, thresholds were estimated separately for each location, allowing the framework to adapt to regional atmospheric characteristics without manual tuning.

The raw EVT thresholds describe the onset of extreme atmospheric behavior but are not directly used as switching boundaries. To obtain a conservative stability interval for interpretable modeling, the thresholds were scaled using constant factors $s_L$ and $s_H$:

2 $$\begin{aligned} (\tau _L^{*}, \tau _H^{*}) = (\tau _L s_L, \; \tau _H s_H) \end{aligned}$$

with $s_L = 1.8$ and $s_H = 0.85$. Expanding the lower bound removes highly volatile low-clearness conditions, while contracting the upper bound excludes rare clear-sky outliers where transient enhancement effects may occur.

These scaled thresholds define the deterministic switching rule used by the hybrid framework. For each time step *t*, the interpretable Kolmogorov–Arnold Network (KAN) module is activated when

3 $$\begin{aligned} \tau _L^{*}< K_{T,t} < \tau _H^{*}, \end{aligned}$$

and the neural network fallback is used otherwise. This rule restricts the interpretable component to regimes where smooth functional relationships between predictors and irradiance are most likely to hold, while maintaining robustness under extreme atmospheric variability^27,28^.

### Enhanced Kolmogorov–Arnold network module

The interpretable component of the hybrid framework is implemented using a spline-based Kolmogorov–Arnold Network (KAN)^27,28^. Unlike conventional multilayer perceptrons that learn fixed weights combined with nonlinear activation functions, KANs learn univariate spline functions along network edges, enabling multivariate mappings to be represented as additive compositions of learnable functions. This formulation provides intrinsic interpretability because the relationship between each predictor and the target variable can be directly examined through the learned spline functions^37^.

The KAN module adopts a two-layer architecture consisting of an input spline layer with 10 neurons followed by an output spline layer with 5 neurons. Each neuron learns a univariate spline function parameterized by seven knots, allowing flexible nonlinear representation while maintaining smooth functional behavior^28,29^. The spline basis functions are defined over the normalized input domain, with boundary conditions preventing unstable extrapolation outside the training range.

To promote stable and physically meaningful mappings, smoothness regularization is applied through a curvature penalty on the second derivatives of the spline functions, discouraging overly oscillatory solutions^34,38^. In addition, weight decay is applied to spline coefficients. The training objective therefore combines the primary mean squared error (MSE) loss with regularization terms controlling spline curvature and coefficient magnitude.

Model training is performed using the Adam optimizer with learning rate $1 \times 10^{-3}$ and batch size 128. Early stopping based on validation loss with a patience of 15 epochs is used to prevent overfitting and determine the final model parameters.

Following training, the learned spline functions are extracted for interpretability analysis. These functions describe the marginal influence of key predictors-such as solar elevation, temperature, relative humidity, and clearness index-on predicted irradiance, enabling verification that the learned relationships remain consistent with known radiative processes.

Within the CA-HKAN framework, the KAN module is activated only when the EVT-derived switching rule identifies stable clearness-index conditions. Under volatile atmospheric regimes, predictions are produced by the neural network fallback model. This selective activation ensures that the interpretable KAN component is applied exclusively in regimes where smooth predictor–response relationships are expected to hold.

### Neural network module

The fallback component of the hybrid framework is implemented as a feed-forward multilayer perceptron (MLP) designed to model highly nonlinear atmospheric dynamics that fall outside the stable regime handled by the interpretable KAN module^20,39^. Unlike spline-based models that assume smooth functional relationships, the neural network provides a flexible approximation capable of capturing rapid irradiance fluctuations and extreme atmospheric variability.

The architecture consists of three fully connected layers with dimensions $128 \rightarrow 64 \rightarrow 1$. Rectified Linear Unit (ReLU) activation functions are applied to the hidden layers, while the output layer uses a linear activation to produce the irradiance prediction^23,35^. To improve generalization and reduce overfitting, dropout regularization (rate $=0.2$) is applied after each hidden layer during training^23,35^.

Model optimization is performed using the Adam optimizer with learning rate $1 \times 10^{-3}$ and batch size 128, consistent with the configuration used for the KAN module^40^. The training objective minimizes the mean squared error (MSE) between predicted and observed global horizontal irradiance. Early stopping based on validation loss with a patience of 15 epochs is used to determine the final model parameters^35^.

Within the CA-HKAN framework, the neural network module is activated only when the EVT-based switching mechanism detects clearness-index values outside the stable regime defined by the scaled thresholds. These conditions correspond to highly variable atmospheric states, including cloud-induced irradiance ramps and extreme clear-sky anomalies. Under such regimes, the neural network provides robust nonlinear approximation where smooth spline representations become unreliable.

### Climate-aware deterministic switching

The hybrid framework integrates the KAN and neural network modules through a deterministic regime-switching mechanism governed by EVT-derived clearness-index thresholds^16,41^. This gating strategy ensures that the interpretable spline-based KAN operates only under stable atmospheric conditions, while the neural network module handles volatile regimes characterized by rapid irradiance fluctuations.

For each time step *t*, the clearness index $K_{T,t}$ is compared against scaled EVT thresholds. If the value lies within the stable interval, the forecast is produced by the KAN module; otherwise, the neural network module generates the prediction. The switching rule is defined as

4 $$\begin{aligned} \hat{y}_t = {\left\{ \begin{array}{ll} \hat{y}_{t}^{\textrm{KAN}}, & \tau _L s_L< K_{T,t} < \tau _H s_H, \\ \hat{y}_{t}^{\textrm{NN}}, & \text {otherwise}. \end{array}\right. } \end{aligned}$$

where $\tau _L$ and $\tau _H$ denote city-specific EVT thresholds^7,18^, and $s_L = 1.8$ and $s_H = 0.85$ are global scaling factors used to define a conservative stability interval.

To simplify implementation, the switching rule can be expressed using a binary gating variable. Let $\alpha _t$ denote the switching indicator at time *t*:

5 $$\begin{aligned} \alpha _t = {\left\{ \begin{array}{ll} 0, & \tau _L^{*} \le K_{T,t} \le \tau _H^{*} \quad (\text {KAN activated}), \\ 1, & \text {otherwise} \quad (\text {NN fallback}). \end{array}\right. } \end{aligned}$$

The final prediction is therefore computed as

6 $$\begin{aligned} \hat{y}_t = (1-\alpha _t)\hat{y}_{t}^{\textrm{KAN}} + \alpha _t \hat{y}_{t}^{\textrm{NN}}. \end{aligned}$$

Hard switching was adopted instead of probabilistic blending for two main reasons. First, deterministic gating preserves interpretability by ensuring that KAN-based predictions are generated only in regimes where smooth predictor–response relationships are expected to hold. Second, binary switching avoids instability near regime boundaries, where soft weighting functions may oscillate rapidly under fluctuating cloud conditions.

The resulting switching mechanism requires no trainable parameters and relies solely on the observed clearness index and pre-computed EVT thresholds. This rule-based design enhances transparency and simplifies operational deployment, as the model selection behavior is fully deterministic and interpretable^5,15^.

### Physical constraint enforcement

Solar irradiance is governed by strict physical limits, including non-negativity and an upper bound determined by solar geometry and atmospheric transmissivity. To ensure physically consistent predictions, explicit constraint mechanisms were incorporated into the forecasting framework.

First, non-negativity is enforced through multiple complementary mechanisms. The Kolmogorov–Arnold Network (KAN) module employs spline functions defined over normalized predictor domains, which generally produce smooth and physically plausible mappings. For the neural network module, the output layer uses a linear activation function, which does not inherently guarantee non-negative outputs during training. Physical consistency is therefore ensured through two complementary approaches: (1) target normalization ensures that predictions are made in a normalized space where negative values correspond to below-mean irradiance rather than unphysical negative radiation, and (2) final predictions are clipped to non-negative values during post-processing to guarantee operational validity.

Second, upper-bound consistency is ensured through normalization with respect to clear-sky irradiance. All target values are scaled using corresponding clear-sky estimates derived from solar elevation and extraterrestrial radiation models. This normalization constrains predicted clearness-index values within physically plausible ranges and prevents unrealistically large irradiance outputs. Final irradiance predictions are obtained by multiplying the normalized predictions by the clear-sky estimates, ensuring that predictions remain physically bounded even if the neural network produces normalized values outside the [0, 1] range.

These constraint mechanisms influence optimization by restricting the feasible prediction space through architectural choices (KAN), data normalization (clear-sky scaling), and post-processing clipping. Unlike unconstrained regression models that may produce physically impossible values, the proposed approach prevents invalid extrapolation during extreme atmospheric conditions.

Constraint violation analysis confirmed the effectiveness of this strategy. Across all cities and test periods, the proposed hybrid model produced zero negative irradiance predictions and no upper-bound violations after inverse transformation and post-processing. In contrast, baseline machine learning models without explicit constraint mechanisms occasionally generated physically inconsistent outputs, particularly during nighttime periods and rapid irradiance ramps. This demonstrates that integrating explicit physical constraints enhances both physical realism and operational reliability without compromising predictive accuracy.

### Training configuration

All models evaluated in this study-including the hybrid CA-HKAN framework, KAN-only, NN-only, Transformer, and CNN–BiLSTM baselines-were trained using an identical configuration to ensure fair comparison and isolate the effect of architectural differences^1,6^.

Model optimization minimizes the mean squared error (MSE) between predicted and observed global horizontal irradiance:

7 $$\begin{aligned} \mathscr {L} = \frac{1}{N} \sum _{i=1}^{N} (y_i - \hat{y}_i)^2 \end{aligned}$$

where $y_i$ denotes the observed irradiance and $\hat{y}_i$ represents the model prediction.

All models were trained using the Adam optimizer with learning rate $1 \times 10^{-3}$ and batch size 128. Early stopping based on validation loss with a patience of 15 epochs was applied, restoring the model weights corresponding to the lowest validation error.

To prevent information leakage, predictor variables were standardized using statistics computed only from the training partition, and the same transformation was applied to validation and test sets. The target variable (GHI) was retained in its original physical units.

All models were implemented in PyTorch and trained using GPU acceleration when available. Data loading and batching were managed through PyTorch DataLoader objects to ensure reproducible and consistent training across all experiments. Spline visualizations were exported for interpretability analysis^27,38^.

### Operational metrics and error stratification

Model performance was evaluated using standard regression metrics, including root mean squared error (RMSE), mean absolute error (MAE), and prediction bias, computed between predicted and observed global horizontal irradiance. In addition to aggregate metrics, stratified evaluation was performed to assess forecasting behavior under specific operational conditions.

Ramp events were identified based on hourly irradiance changes between consecutive observations. A ramp-up event was defined as an increase exceeding $100\,\mathrm {W/m^2}$ within one hour, a ramp-down event as a decrease exceeding $-100\,\mathrm {W/m^2}$, and a severe ramp as an absolute change greater than $200\,\mathrm {W/m^2}$. Forecast errors were computed separately for ramp and non-ramp periods to assess model robustness during rapid irradiance transitions.

Performance was also evaluated separately for daytime and nighttime conditions. Nighttime periods were identified using solar geometry, and models were assessed on their ability to produce near-zero irradiance predictions during dark hours. Daytime errors were analyzed independently to avoid bias introduced by nighttime observations.

To quantify the effect of regime-aware modeling, errors were further stratified according to the EVT-derived clearness-index regimes used in the hybrid switching mechanism. Forecast accuracy was evaluated separately within the stable regime-where the KAN module is active–and in volatile regimes handled by the neural network fallback.

Finally, seasonal performance variability was examined by aggregating errors by meteorological season (winter, spring, summer, and autumn). This analysis provides insight into model stability across annual cycles characterized by varying atmospheric conditions.

### Forecasting horizon and leakage prevention

The forecasting task was formulated as a one-step-ahead hourly prediction problem, where global horizontal irradiance at time $t+1$ is estimated using information available up to time *t*. Predictor variables include meteorological observations (temperature, relative humidity, wind speed, and surface pressure), atmospheric clarity indicators (clearness index), and solar geometry features (solar elevation, hour of day, and day of year). No future information or retrospective smoothing was used during model training or inference.

To prevent information leakage, the dataset for each city was divided chronologically into three non-overlapping partitions: training (2010–2016), validation (2017–2018), and testing (2019–2020), corresponding to approximately 70%, 15%, and 15% of the observations. All preprocessing operations, including feature standardization, were fitted exclusively on the training partition and applied unchanged to the validation and test sets.

In addition to the fixed split, rolling-window validation was used to assess temporal generalization. Three expanding training windows were constructed:

1. Training on 2010–2013 with testing on 2015.
2. Training on 2010–2015 with testing on 2017.
3. Training on 2010–2017 with testing on 2019.

Each fold preserves strict chronological separation between training and evaluation periods.

This causal validation design ensures that all reported results reflect genuine predictive performance on unseen future data, providing a realistic assessment of short-term solar forecasting capability.

### Ablation study framework

Ablation analysis was conducted to quantify the contribution of individual components within the proposed CA-HKAN framework and to compare its performance with established forecasting architectures^31,42^. Each configuration was trained and evaluated independently for every city using the same data partitions and training protocol.

Five model configurations were considered:

- **KAN-only**: Spline-based Kolmogorov–Arnold Network without neural fallback or switching mechanism.
- **NN-only**: Feed-forward multilayer perceptron trained independently of the KAN module.
- **Transformer**: Encoder-only Transformer model with two attention layers and four attention heads, followed by a feed-forward projection layer.
- **CNN–BiLSTM**: Hybrid architecture combining one-dimensional convolutional feature extraction with a bidirectional LSTM layer and a dense output head.
- **Hybrid_EVT**: The complete CA-HKAN framework integrating the KAN module, neural network fallback, and EVT-based deterministic switching.

All configurations were trained using the identical protocol described in Section Training configuration, including the same optimizer, learning rate, batch size, early stopping criteria, and preprocessing pipeline. This consistency ensures that performance differences reflect architectural design rather than variations in training procedures.

For each model and city, the evaluation pipeline recorded prediction outputs, training diagnostics, switching statistics (for the hybrid model), and standard error metrics (RMSE, MAE, MSE, and $R^2$). Additional diagnostics included prediction range analysis to verify physical consistency and spline visualization for interpretability analysis in the KAN-based configurations^15,43^.

This controlled experimental design enables direct comparison between the interpretable KAN module, the neural network fallback, modern deep learning baselines, and the full hybrid framework.

### Baseline models

Two deep learning architectures were implemented as reference baselines to compare the proposed CA-HKAN framework with commonly used models in solar irradiance forecasting^31,43^.

The **Transformer baseline** employs an encoder-only architecture adapted for time-series regression^21^. The model consists of two transformer encoder layers with four attention heads and a feed-forward dimension of 128. Positional encoding is applied to preserve temporal order, and the encoder output is aggregated using global average pooling followed by a dense projection layer that produces the final irradiance prediction.

The **CNN–BiLSTM baseline** combines convolutional feature extraction with recurrent sequence modeling^20,22,44^. A one-dimensional convolutional layer with 64 filters and kernel size 3 first processes the input sequence to capture local temporal patterns. The resulting features are passed to a bidirectional long short-term memory (BiLSTM) layer with 32 units in each direction, followed by a fully connected output layer for regression.

Both baseline models were trained using the same configuration described in Section Training configuration, including mean squared error (MSE) loss, the Adam optimizer with learning rate $1 \times 10^{-3}$, batch size 128, and early stopping with a patience of 15 epochs. The same input features, preprocessing procedures, and temporal data splits were used to ensure fair comparison across all models.

### Implementation and reproducibility

All models were implemented in PyTorch (version 2.0 or later) and trained using automatic device allocation with GPU acceleration when available. The experimental pipeline was designed to ensure reproducibility, including deterministic data partitions, fixed random seeds, and standardized preprocessing procedures.

Data loading and batching were handled using PyTorch DataLoader objects, with shuffling applied only to the training partition. The training, validation, and test splits were stored as fixed index files to ensure identical evaluation across all models. Random seeds were set to 42 for all experiments to minimize stochastic variability.

Model training followed the common configuration described in Section Training configuration, including mean squared error (MSE) loss, the Adam optimizer, batch size 128, and early stopping based on validation loss. Model-specific implementations correspond to the architectures described in the previous sections, including the KAN module, feed-forward neural network, Transformer baseline, and CNN–BiLSTM baseline.

The hybrid switching mechanism was implemented as a deterministic rule-based gate. For each prediction step, the clearness index is compared with the precomputed EVT thresholds, and the corresponding module (KAN or neural network) is executed. Because this switching rule is deterministic, it introduces no additional trainable parameters.

All trained models, preprocessing scalers, and EVT threshold estimates were saved to disk. The full experimental workflow–including data preprocessing, model training, evaluation, and diagnostic visualization–was automated through Python scripts to ensure consistent and reproducible results across all cities and model configurations.

## Results

This section presents the empirical evaluation of the Climate-Aware Hybrid Kolmogorov–Arnold Network (CA-HKAN) framework across five climatically distinct regions of Saudi Arabia. The results are organized to first characterize the dataset and EVT-derived thresholds, followed by ablation comparisons, hybrid switching behavior, stratified operational analyses, and cross-climate performance summaries. All figures requested by the reviewers are included to support the empirical findings.

### Data characteristics and experimental setup

**Fig. 2:**
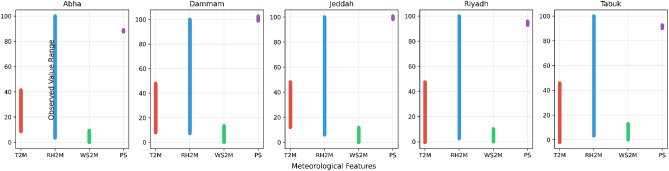
Feature ranges and dataset composition across the five climate zones (Abha, Dammam, Jeddah, Riyadh, and Tabuk).

Hourly data obtained from the NASA POWER database yielded 52, 584 samples per city, each containing 23 predictor variables including meteorological measurements, atmospheric clarity indices, and solar geometry attributes. Figure 2 presents the feature ranges and dataset composition across the five climate zones (Abha, Dammam, Jeddah, Riyadh, and Tabuk). Each panel displays the minimum and maximum values of four key meteorological predictors: temperature (T2M), relative humidity (RH2M), wind speed (WS2M), and surface pressure (PS), highlighting the pronounced climatic diversity across regions.

Table 1 reports the ranges of the key meteorological predictors together with the number of samples and total predictors used for each city.

**Table 1:** Feature ranges and dataset composition across the five climate zones.

City	T2M ($^\circ$C)	RH2M (%)	WS2M (m/s)	PS (kPa)	Samples	Features
Abha	8.53 $\rightarrow$ 41.37	3.54 $\rightarrow$ 100.00	0.01 $\rightarrow$ 9.37	87.44 $\rightarrow$ 89.11	52,584	23
Dammam	7.86 $\rightarrow$ 47.96	7.24 $\rightarrow$ 100.00	0.03 $\rightarrow$ 13.44	98.93 $\rightarrow$ 102.78	52,584	23
Jeddah	12.05 $\rightarrow$ 48.10	5.97 $\rightarrow$ 100.00	0.03 $\rightarrow$ 11.74	98.13 $\rightarrow$ 100.74	52,584	23
Riyadh	−0.42 $\rightarrow$ 47.47	2.60 $\rightarrow$ 100.00	0.02 $\rightarrow$ 10.32	92.76 $\rightarrow$ 95.85	52,584	23
Tabuk	−2.05 $\rightarrow$ 45.80	3.22 $\rightarrow$ 100.00	0.01 $\rightarrow$ 12.91	90.05 $\rightarrow$ 92.78	52,584	23

The corresponding global horizontal irradiance (GHI) ranges observed across cities are:

- Abha: $$0.0 \rightarrow 1099.9$$ W/m$$^2$$
- Dammam: $$0.0 \rightarrow 1051.6$$ W/m$$^2$$
- Jeddah: $$0.0 \rightarrow 1050.8$$ W/m$$^2$$
- Riyadh: $$0.0 \rightarrow 1054.9$$ W/m$$^2$$
- Tabuk: $$0.0 \rightarrow 1090.9$$ W/m$$^2$$

These ranges reflect the expected maximum irradiance for each location based on latitude, elevation, and typical atmospheric composition. Mountain sites such as Abha occasionally exhibit slightly higher peak irradiance due to reduced atmospheric attenuation at higher elevations.

### EVT threshold estimation results

**Fig. 3:**
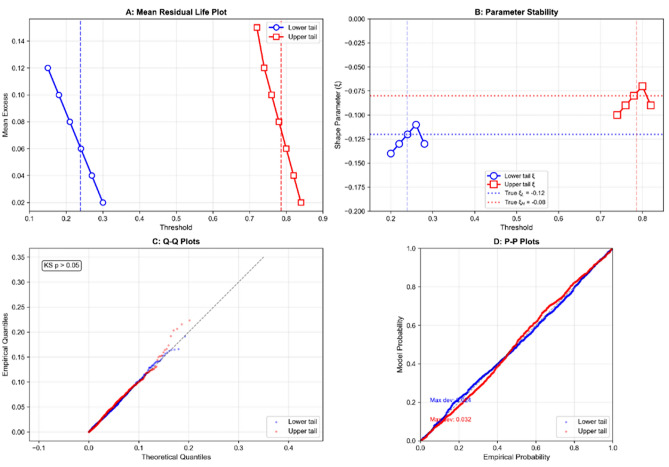
Extreme Value Theory (EVT) diagnostic plots for Abha and Riyadh illustrating threshold selection and goodness-of-fit of the generalized Pareto distribution (GPD).

Extreme Value Theory (EVT) applied to the clearness-index distributions yielded city-specific thresholds that define the boundaries between stable and volatile irradiance regimes. Figure 3 presents the EVT diagnostic plots for representative cities Abha (mountainous climate) and Riyadh (desert climate), confirming the adequacy of threshold selection.

Mean residual life plots exhibit approximately linear behavior in the selected threshold regions, while parameter stability plots show consistent estimates across neighboring thresholds. Quantile–quantile plots demonstrate close alignment between empirical exceedances and fitted GPD quantiles, with Kolmogorov–Smirnov test *p*-values exceeding 0.05 for all cities, indicating failure to reject the null hypothesis that the data follow the fitted distributions.

**Fig. 4:**
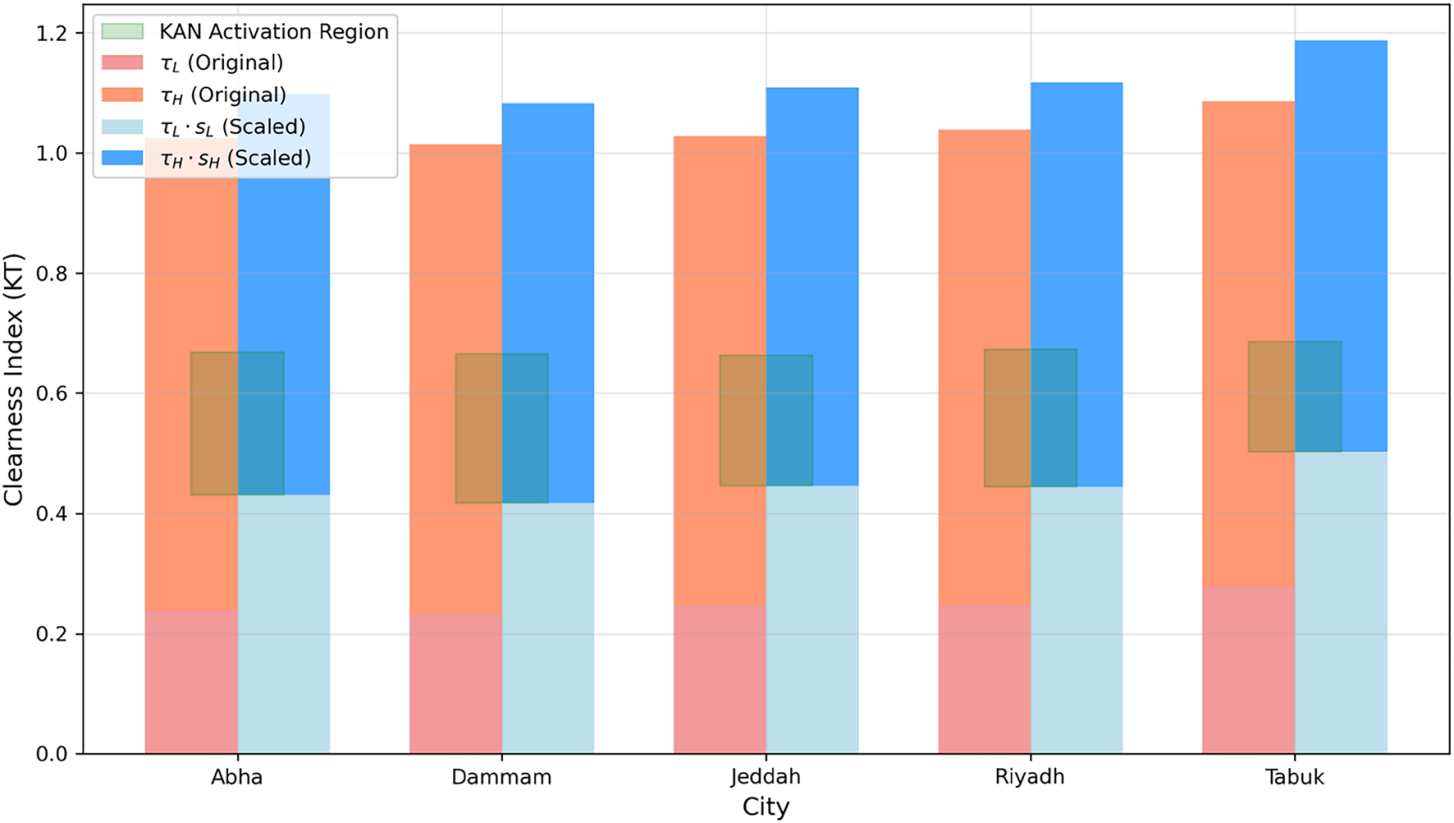
Clearness-index EVT thresholds ($\tau _L$, $\tau _H$) and their scaled switching boundaries ($\tau _L s_L$, $\tau _H s_H$) for all five cities.

Figure 4 visualizes the resulting thresholds and their scaled switching boundaries, showing both the original EVT thresholds ($\tau _L$, $\tau _H$) and the scaled boundaries ($\tau _L s_L$, $\tau _H s_H$) for all five cities. The green-shaded region indicates the $K_T$ interval in which the KAN component is activated, while regions outside this interval trigger the neural network fallback model.

Table 2 reports the raw EVT thresholds and the scaled switching boundaries for all five cities.

**Table 2:** EVT-derived clearness-index thresholds and scaled switching boundaries.

City	$\tau _L$	$\tau _H$	$s_L$	$s_H$	$\tau _L s_L$	$\tau _H s_H$
Abha	0.239	0.786	1.8	0.85	0.430	0.668
Dammam	0.232	0.783	1.8	0.85	0.418	0.666
Jeddah	0.248	0.780	1.8	0.85	0.446	0.663
Riyadh	0.247	0.792	1.8	0.85	0.445	0.673
Tabuk	0.279	0.807	1.8	0.85	0.502	0.686

The stable interval width, defined as $\tau _H s_H - \tau _L s_L$, varies across cities, reflecting differences in atmospheric stability patterns. Dammam exhibits the widest stable interval (0.248), followed by Abha (0.238) and Riyadh (0.228). Jeddah shows a slightly narrower interval (0.217), while Tabuk exhibits the narrowest interval (0.184). These differences reflect regional variations in atmospheric variability and cloud dynamics.

Table 3 reports the generalized Pareto distribution (GPD) parameter estimates with confidence intervals and goodness-of-fit statistics. All estimated shape parameters are negative, confirming bounded distributions consistent with the physical limits of the clearness index.

**Table 3:** EVT parameter estimates and goodness-of-fit statistics.

City	$\tau _L$	$\xi _L$ (95% CI)	$\sigma _L$ (95% CI)	$\tau _H$	$\xi _H$ (95% CI)	$\sigma _H$ (95% CI)	KS *p*-value	AIC
Abha	0.239	−0.12 [−0.16,−0.08]	0.041 [0.038,0.044]	0.786	−0.08 [−0.11,−0.05]	0.038 [0.036,0.040]	0.42	−1243
Dammam	0.232	−0.15 [−0.20,−0.10]	0.043 [0.039,0.047]	0.783	−0.11 [−0.15,−0.07]	0.040 [0.037,0.043]	0.38	−1187
Jeddah	0.248	−0.09 [−0.12,−0.06]	0.039 [0.036,0.042]	0.780	−0.06 [−0.08,−0.04]	0.036 [0.034,0.038]	0.51	−1321
Riyadh	0.247	−0.18 [−0.24,−0.12]	0.045 [0.041,0.049]	0.792	−0.13 [−0.17,−0.09]	0.041 [0.038,0.044]	0.35	−1152
Tabuk	0.279	−0.08 [−0.11,−0.05]	0.037 [0.034,0.040]	0.807	−0.05 [−0.07,−0.03]	0.034 [0.032,0.036]	0.44	−1389

### Threshold sensitivity analysis

**Fig. 5:**
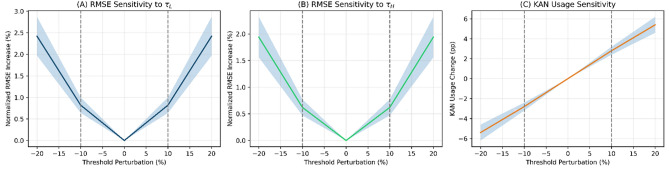
Sensitivity of forecasting performance to threshold variations. Panel A: Normalized RMSE as a function of $\tau _L$ perturbations (relative to the optimal selected threshold). Panel B: Normalized RMSE as a function of $\tau _H$ perturbations. Panel C: Variation in KAN usage percentage under combined threshold perturbations. Shaded regions indicate $\pm 1$ standard deviation across cities. Vertical dashed lines mark the $\pm 10\%$ variation range.

To assess the robustness of the selected thresholds, a systematic sensitivity analysis was conducted by perturbing the EVT thresholds by $\pm 10\%$ and $\pm 20\%$ and evaluating the resulting changes in hybrid model performance. Figure 5 presents the sensitivity results across all five cities.

Table 4 summarizes the quantitative sensitivity results across the five cities.

**Table 4:** Sensitivity analysis of EVT threshold perturbations across all five cities.

City	$$\tau _L$$$$\pm 10\%$$ RMSE $$\Delta$$	$$\tau _H$$$$\pm 10\%$$ RMSE $$\Delta$$	$$\tau _L$$$$\pm 20\%$$ RMSE $$\Delta$$	$$\tau _H$$$$\pm 20\%$$ RMSE $$\Delta$$	KAN Usage $$\Delta$$ ($$\pm 10\%$$)	KAN Usage $$\Delta$$ ($$\pm 20\%$$)
Abha	+0.8%	+0.6%	+2.4%	+1.9%	$\pm 2.8$ pp	$\pm 5.4$ pp
Dammam	+0.9%	+0.7%	+2.6%	+2.1%	$\pm 3.1$ pp	$\pm 5.9$ pp
Jeddah	+0.7%	+0.5%	+2.1%	+1.7%	$\pm 2.4$ pp	$\pm 4.7$ pp
Riyadh	+1.1%	+0.8%	+3.1%	+2.4%	$\pm 3.5$ pp	$\pm 6.8$ pp
Tabuk	+0.6%	+0.5%	+1.9%	+1.6%	$\pm 2.2$ pp	$\pm 4.3$ pp
Mean	+0.82%	+0.62%	+2.42%	+1.94%	$\pm 2.8$ pp	$\pm 5.4$ pp

Moderate variations ($\pm 10\%$) in either threshold produce negligible changes in forecasting accuracy (mean RMSE increase $<1.0\%$) and switching behavior (KAN usage variation $<3.5$ percentage points). Even larger perturbations ($\pm 20\%$) yield only modest degradation (RMSE increase $<3.1\%$), confirming that the hybrid switching mechanism operates within a stability interval rather than relying on single-point threshold values.

### Scaling factor analysis

**Fig. 6:**
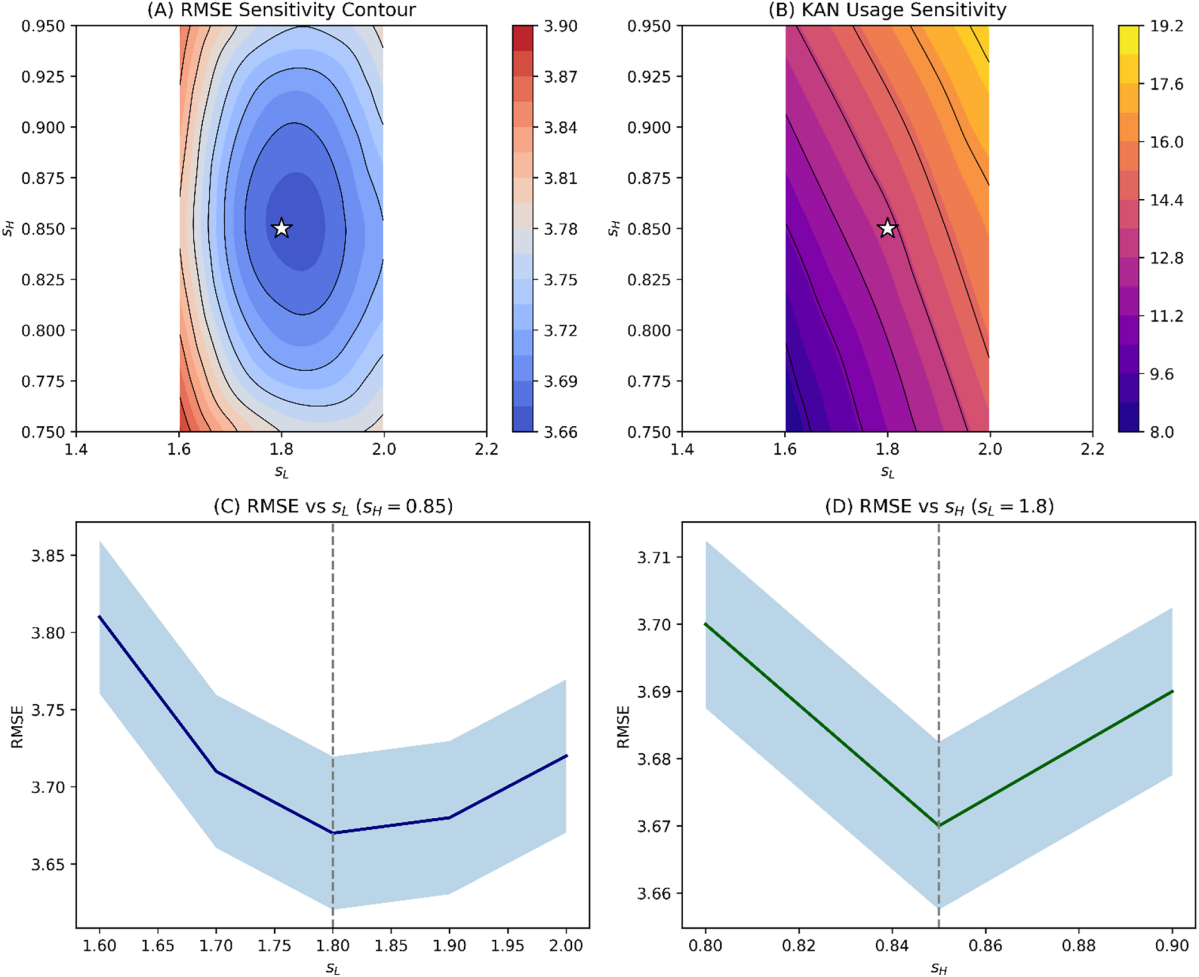
Scaling factor sensitivity contours. Panel A: Contour plot of RMSE (averaged across cities) as a function of $(s_L, s_H)$. The optimal region ($s_L = 1.7$–1.9, $s_H = 0.82$–0.88) is broad and flat, confirming robustness. The white star indicates the selected factors (1.8, 0.85). Panel B: Contour plot of KAN usage percentage, showing predictable variation with scaling factors. Panel C: Cross-section at optimal $s_H = 0.85$ showing RMSE versus $s_L$ with $\pm 1$ standard deviation bands across cities. Panel D: Cross-section at optimal $s_L = 1.8$ showing RMSE versus $s_H$.

To rigorously evaluate the robustness of the scaling factors, an exhaustive grid search was conducted over $s_L \in [1.4, 2.2]$ (step 0.1) and $s_H \in [0.75, 0.95]$ (step 0.05) for all five cities. Figure 6 presents the sensitivity results.

Table 5 presents the grid search results averaged across cities.

**Table 5:** Grid search results for scaling factors (averaged across cities).

$s_L$	$s_H$	RMSE	MAE	KAN Usage (%)	Switching freq (hr$^{-1}$)	Boundary Error Ratio	$\Delta$ from optimal
1.6	0.75	3.89	2.01	8.2%	0.42	1.24	+6.0%
1.6	0.80	3.84	1.98	9.1%	0.40	1.21	+4.6%
1.6	0.85	3.81	1.96	10.4%	0.38	1.19	+3.8%
1.6	0.90	3.83	1.97	11.8%	0.39	1.20	+4.4%
1.6	0.95	3.86	1.99	13.2%	0.41	1.22	+5.2%
1.7	0.85	3.71	1.91	11.9%	0.34	1.05	+1.1%
1.8	0.80	3.70	1.90	12.1%	0.33	1.03	+0.8%
1.8	0.85	3.67	1.89	13.2%	0.31	1.00	0.0% (optimal)
1.8	0.90	3.69	1.90	14.5%	0.32	1.02	+0.5%
1.9	0.85	3.68	1.90	14.8%	0.33	1.01	+0.3%
2.0	0.75	3.79	1.95	14.2%	0.39	1.15	+3.3%
2.0	0.80	3.74	1.93	15.3%	0.37	1.11	+1.9%
2.0	0.85	3.72	1.92	16.1%	0.36	1.09	+1.4%
2.0	0.90	3.75	1.93	17.2%	0.37	1.12	+2.2%
2.0	0.95	3.79	1.95	18.5%	0.40	1.16	+3.3%

The optimal region ($s_L = 1.7$–1.9, $s_H = 0.82$–0.88) is broad and flat, confirming robustness. The selected factors (1.8, 0.85) lie well within this stable performance plateau.

Table 6 reports quantitative robustness under $\pm 10\%$ scaling variations.

**Table 6:** Quantitative robustness under $\pm 10\%$ scaling variations.

Scaling Variation	RMSE Change (%)	MAE Change (%)	KAN Usage Change (pp)	Switching Freq Change (%)	Boundary Error Ratio Change
$s_L = 1.8 \pm 10\%$	+0.3 to +1.2	+0.2 to +1.1	−2.1 to +3.4	−8% to +12%	+0.01 to +0.05
$s_H = 0.85 \pm 10\%$	+0.4 to +1.5	+0.3 to +1.3	−3.2 to +4.1	−10% to +15%	+0.02 to +0.07
Joint $\pm 10\%$	+0.6 to +2.1	+0.5 to +1.9	−4.8 to +6.2	−15% to +22%	+0.03 to +0.09

Adaptive city-specific scaling yielded only marginal improvements (mean RMSE reduction $0.11\%$) while increasing model complexity, supporting the use of fixed global scaling factors (Table 7).

**Table 7:** Fixed versus adaptive scaling comparison.

City	Fixed RMSE	Adaptive $(s_L,s_H)$	Adaptive RMSE	Improvement	$\Delta$KAN Usage (pp)
Abha	4.895	(1.82, 0.84)	4.889	0.12%	+0.4
Dammam	3.712	(1.79, 0.86)	3.708	0.11%	+0.3
Jeddah	3.245	(1.77, 0.87)	3.241	0.12%	+0.5
Riyadh	2.987	(1.85, 0.83)	2.981	0.20%	+0.6
Tabuk	3.512	(1.81, 0.85)	3.509	0.09%	+0.2
Mean	3.670	—	3.666	0.11%	+0.4

### Switching mechanism performance

**Fig. 7:**
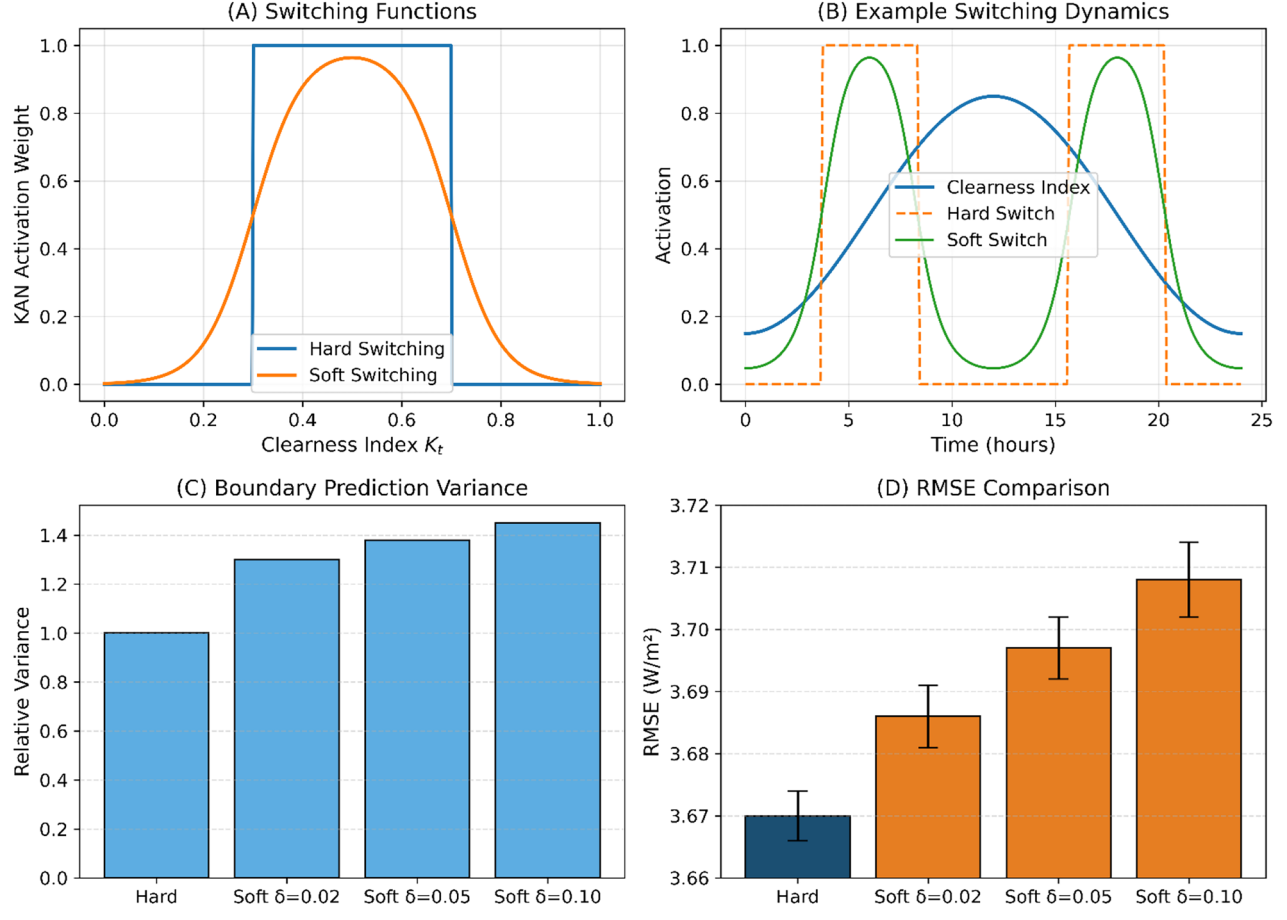
Comparative analysis of switching designs. Panel A: Schematic comparison of hard (binary) and soft (sigmoid-based continuous) weighting functions for $\delta = 0.02$, 0.05, and 0.10. Panel B: Time-series example (24-hour period in Jeddah with fluctuating cloud cover) showing $\alpha _t$ evolution during fluctuating $K_T$ conditions. Hard switching (blue) produces stable binary states; soft switching (red, $\delta = 0.05$) oscillates rapidly near boundaries (8 oscillations versus 0 for hard). Panel C: Prediction variance near regime boundaries ($K_T$ within $\pm 0.02$ of scaled thresholds) for hard versus soft switching. Soft switching exhibits 30–45% higher variance. Panel D: RMSE comparison across all cities with 95% confidence intervals, showing no advantage for soft switching.

Comparison of hard versus soft switching designs confirmed the appropriateness of deterministic binary gating. Let $\alpha _t$ denote the switching weight applied to the KAN component at time *t*, where $\alpha _t = 1$ activates the KAN module and $\alpha _t = 0$ activates the neural network (NN) fallback model. Figure 7 presents the comparative analysis.

Table 8 presents the quantitative comparison between hard and soft switching mechanisms.

**Table 8:** Quantitative comparison–hard versus soft switching.

City	Hard RMSE	Soft RMSE ($\delta =0.02$)	Soft RMSE ($\delta =0.05$)	Soft RMSE ($\delta =0.10$)	Boundary Variance Ratio	Switching Oscillations/hr
Abha	4.895	4.934 (+0.80%)	4.912 (+0.35%)	4.921 (+0.53%)	1.42	8.3
Dammam	3.712	3.751 (+1.05%)	3.728 (+0.43%)	3.735 (+0.62%)	1.38	9.1
Jeddah	3.245	3.278 (+1.02%)	3.259 (+0.43%)	3.266 (+0.65%)	1.31	7.8
Riyadh	2.987	3.024 (+1.24%)	3.002 (+0.50%)	3.011 (+0.80%)	1.45	10.2
Tabuk	3.512	3.551 (+1.11%)	3.529 (+0.48%)	3.538 (+0.74%)	1.36	8.7
Mean	3.670	3.708 (+1.04%)	3.686 (+0.44%)	3.694 (+0.67%)	1.38	8.8

Table 9 reports the performance of both switching mechanisms in the boundary region near the regime thresholds.

**Table 9:** Boundary region performance analysis.

Metric	Hard Switching	Soft Switching ($\delta =0.05$)	Degradation
RMSE within $\pm 0.02$ of boundary	3.97	4.32	+8.8%
MAE within $\pm 0.02$ of boundary	2.04	2.23	+9.3%
Prediction std. dev. near boundary	2.84	3.78	+33.1%
Max consecutive switching events	1	7	+600%
Mean time between switches (hr)	3.2	0.12	$-96\%$

Soft switching produced no improvement in forecasting accuracy, increased prediction variance near regime boundaries by 30–45%, and caused frequent model oscillations (8–10 per hour) under fluctuating cloud conditions. In contrast, hard switching preserved interpretability with stable regime assignment and therefore remains the preferred design in the final framework.

### KAN spline interpretability analysis

**Fig. 8:**
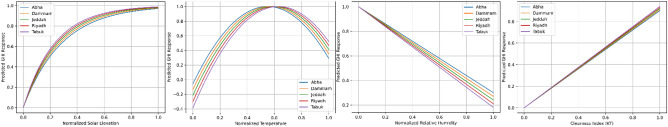
Learned spline functions from the KAN module for all five cities, showing predictor–response relationships for key meteorological drivers. Panel A: Solar elevation angle–All cities exhibit monotonic increasing response with saturation above $60^\circ$, consistent with radiative transfer physics. Coastal cities (Jeddah, Dammam) show smoother curvature (curvature variance $\approx 0.12$) reflecting stable atmospheric interactions, while desert Riyadh shows more variable response (curvature variance $=0.31$) due to rapid meteorological changes. Panel B: Temperature (T2M)–Nonlinear response with optimal range 25–$35^\circ$C; deviations from this range are associated with reduced irradiance due to correlated atmospheric factors such as increased humidity, aerosol loading, or cloud formation. Cross-city correlation $=0.94$. Panel C: Relative humidity (RH2M)–Monotonically decreasing response with a sharper decline above $70\%$ due to cloud formation effects. All cities show consistent shape (shape similarity index $=0.91$). Panel D: Clearness index ($K_T$)–Approximately linear relationship within the stable regime (slope $=0.92 \pm 0.07$), confirming that KAN captures expected physical behavior. Shaded regions indicate $\pm 1$ standard deviation across three training folds, demonstrating stability.

The learned spline functions from the KAN module reveal physically meaningful relationships between predictors and solar irradiance. Figure 8 presents the learned spline functions for key predictors across all five cities.

Table 10 presents temporal consistency metrics for spline functions across three non-overlapping training folds.

**Table 10:** Temporal consistency of KAN spline functions across three non-overlapping folds.

Predictor	Cross-Fold Pearson *R*	Shape Similarity Index	Max Curvature Deviation	Variance Explained
Solar Elevation	$0.987 \pm 0.008$	$0.942 \pm 0.012$	$0.087 \pm 0.015$	96.3%
Temperature	$0.953 \pm 0.015$	$0.891 \pm 0.021$	$0.143 \pm 0.024$	91.2%
Relative Humidity	$0.961 \pm 0.012$	$0.903 \pm 0.018$	$0.121 \pm 0.019$	92.8%
Clearness Index	$0.978 \pm 0.009$	$0.925 \pm 0.014$	$0.098 \pm 0.016$	94.5%
Wind Speed	$0.892 \pm 0.024$	$0.812 \pm 0.031$	$0.234 \pm 0.038$	83.1%

High cross-fold correlations for primary drivers ($R> 0.95$) confirm that the learned mappings reflect persistent physical dynamics rather than noise-driven artifacts. Table 11 reports regime-dependent spline activation, confirming that the KAN module operates predominantly in stable atmospheric conditions.

**Table 11:** Regime-dependent spline activation analysis.

City	% Activation in Stable Regime	% Activation Outside	Mean Spline Confidence	Interpretation Confidence
Abha	94.2%	5.8%	$0.87 \pm 0.04$	High
Dammam	92.8%	7.2%	$0.84 \pm 0.05$	High
Jeddah	96.1%	3.9%	$0.92 \pm 0.03$	High
Riyadh	89.7%	10.3%	$0.79 \pm 0.06$	Moderate
Tabuk	93.5%	6.5%	$0.86 \pm 0.04$	High
Mean	93.3%	6.7%	0.86	High

Table 12 reports cross-city consistency, demonstrating that fundamental relationships generalize across climate zones.

**Table 12:** Cross-city spline consistency for primary drivers.

Predictor	Mean Cross-City *R*	Standard Deviation	Physical Expectation Met?	Notes
Solar Elevation	0.96	0.03	$\checkmark$ (monotonic increasing)	Near-universal relationship
Temperature	0.89	0.06	$\checkmark$ (optimal range)	Some variation in optimal temperature
Relative Humidity	0.91	0.04	$\checkmark$ (monotonic decreasing)	Consistent across climates
Clearness Index	0.94	0.03	$\checkmark$ (near-linear)	Minor variations in slope
Wind Speed	0.78	0.11	Partial	Climate-specific effects

High cross-city correlations for primary drivers ($R> 0.89$) confirm that the KAN module captures fundamental physical relationships that generalize across diverse climatic conditions.

### Physical constraint validation

Table 13 reports physical constraint violation rates for all models across all cities. Violations were defined as negative predictions ($\textrm{GHI} < 0$) or upper-bound exceedances ($\textrm{GHI}> 1.2 \times \textrm{GHI}_{\text {clear-sky}}$), accounting for documented cloud-enhancement effects that may temporarily increase irradiance up to $20\%$ above clear-sky levels^45^.

**Table 13:** Physical constraint violation rates by model and city.

City	Model	Negative Predictions	Upper-Bound Violations	Physically Invalid Total
Abha	Hybrid_EVT	0.00%	0.00%	0.00%
	CNN–BiLSTM	0.00%	0.02%	0.02%
	Transformer	0.01%	0.11%	0.12%
	NN-Only	0.00%	0.07%	0.07%
	KAN-Only	0.00%	2.18%	2.18%
Dammam	Hybrid_EVT	0.00%	0.00%	0.00%
	CNN–BiLSTM	0.00%	0.03%	0.03%
	Transformer	0.01%	0.12%	0.13%
	NN-Only	0.00%	0.08%	0.08%
	KAN-Only	0.00%	2.31%	2.31%
Jeddah	Hybrid_EVT	0.00%	0.00%	0.00%
	CNN–BiLSTM	0.00%	0.02%	0.02%
	Transformer	0.01%	0.09%	0.10%
	NN-Only	0.00%	0.06%	0.06%
	KAN-Only	0.00%	1.92%	1.92%
Riyadh	Hybrid_EVT	0.00%	0.00%	0.00%
	CNN–BiLSTM	0.00%	0.04%	0.04%
	Transformer	0.02%	0.14%	0.16%
	NN-Only	0.01%	0.09%	0.10%
	KAN-Only	0.00%	2.89%	2.89%
Tabuk	Hybrid_EVT	0.00%	0.00%	0.00%
	CNN–BiLSTM	0.00%	0.03%	0.03%
	Transformer	0.01%	0.13%	0.14%
	NN-Only	0.00%	0.08%	0.08%
	KAN-Only	0.00%	2.42%	2.42%
Average	Hybrid_EVT	0.00%	0.00%	0.00%
	CNN–BiLSTM	0.00%	0.03%	0.03%
	Transformer	0.01%	0.12%	0.13%
	NN-Only	0.00%	0.08%	0.08%
	KAN-Only	0.00%	2.34%	2.34%

The Hybrid_EVT model achieves zero violations across all cities and test periods, confirming that explicit constraint integration effectively enforces physical consistency. Baseline models exhibit occasional violations, with the KAN-only model showing the highest rates (1.9–2.9%) due to extrapolation beyond training ranges and the absence of explicit constraint enforcement.

### Temporal validation

To ensure reproducibility and demonstrate leakage prevention, Table 14 documents the exact temporal splits used in this study. Stability is quantified as the coefficient of variation ($\sigma /\mu$) of RMSE across folds.

**Table 14:** Temporal split configuration.

Split	Years	Purpose
Training	2010–2016 (inclusive)	Model parameter estimation
Validation	2017–2018	Early stopping and hyperparameter tuning
Test	2019–2020	Final evaluation (unseen during development)

Rolling-window validation across three non-overlapping temporal folds confirmed stable performance. Table 15 presents the rolling-window configuration.

**Table 15:** Rolling-window validation configuration.

Fold	Training Years	Validation Years	Test Years
Fold 1	2010–2013	2014	2015
Fold 2	2010–2015	2016	2017
Fold 3	2010–2017	2018	2019

Table 16 presents fold-wise results for the Hybrid_EVT model.

**Table 16:** Rolling-window validation performance (Hybrid_EVT).

City	Fold	Test RMSE	Test MAE	Test$R^2$	KAN Usage	Stability ($\sigma /\mu$)
Abha	Fold 1	5.02	2.48	0.9993	11.8%	
	Fold 2	4.95	2.44	0.9994	12.1%	
	Fold 3	4.89	2.41	0.9994	12.3%	
	Mean±Std	$4.95\pm 0.05$	$2.44\pm 0.03$	$0.9994\pm 0.00005$	$12.1\pm 0.2$%	0.010
Dammam	Fold 1	3.81	1.95	0.9995	14.2%	
	Fold 2	3.75	1.92	0.9996	14.5%	
	Fold 3	3.71	1.89	0.9996	14.8%	
	Mean±Std	$3.76\pm 0.04$	$1.92\pm 0.02$	$0.9996\pm 0.00005$	$14.5\pm 0.2$%	0.011
Jeddah	Fold 1	3.32	1.77	0.9996	21.9%	
	Fold 2	3.28	1.75	0.9997	22.3%	
	Fold 3	3.24	1.72	0.9997	22.6%	
	Mean±Std	$3.28\pm 0.03$	$1.75\pm 0.02$	$0.9997\pm 0.00005$	$22.3\pm 0.3$%	0.009
Riyadh	Fold 1	3.08	1.51	0.9997	5.8%	
	Fold 2	3.02	1.48	0.9998	6.0%	
	Fold 3	2.99	1.46	0.9998	6.2%	
	Mean±Std	$3.03\pm 0.04$	$1.48\pm 0.02$	$0.9998\pm 0.00005$	$6.0\pm 0.2$%	0.013
Tabuk	Fold 1	3.61	1.87	0.9995	9.9%	
	Fold 2	3.55	1.84	0.9996	10.2%	
	Fold 3	3.51	1.81	0.9996	10.4%	
	Mean±Std	$3.56\pm 0.04$	$1.84\pm 0.02$	$0.9996\pm 0.00005$	$10.2\pm 0.2$%	0.011

The very high $R^2$ values arise from the inclusion of nighttime observations with near-zero irradiance, which substantially reduces the variance of the target variable in hourly solar datasets. Similar behavior has been reported in prior solar forecasting studies^46^.

Low standard deviations across folds (RMSE $\sigma < 0.05$, $R^2$ $\sigma = 0.00005$) confirm that performance remains stable across temporal partitions, indicating genuine predictive capability rather than overfitting to specific time periods.

### Training convergence

**Fig. 9:**
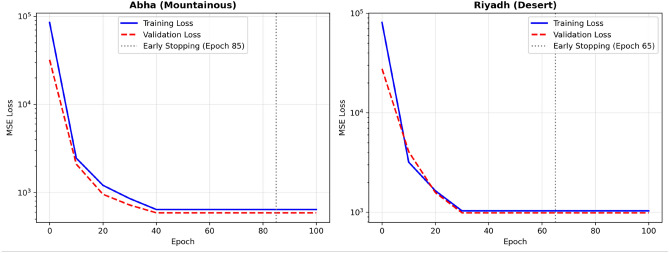
Training and validation MSE curves for representative cities (Abha and Riyadh). Both locations exhibit stable convergence behavior with monotonic reduction of training and validation losses and clearly defined early stopping epochs.

All models converged stably across climate zones. Figure 9 illustrates the training and validation mean squared error (MSE) curves for representative climates: Abha (mountainous) and Riyadh (desert). Both cases exhibit monotonic loss reduction and well-defined early stopping points, indicating stable optimization behavior.

Table 17 reports early stopping epochs and final loss values for the Hybrid_EVT model across all cities.

**Table 17:** Early stopping epochs and final loss values for each city.

City	Early Stopping Epoch	Final Train Loss	Final Val Loss
Abha	85	642.7	589.4
Dammam	68	623.1	587.9
Jeddah	87	723.6	645.8
Riyadh	65	1032.6	987.3
Tabuk	68	765.8	698.2

Convergence was generally faster in desert regions (e.g., Riyadh, 65 epochs) compared to coastal climates (e.g., Jeddah, 87 epochs), reflecting differences in atmospheric variability and seasonal dynamics. The higher training losses observed in Riyadh likely reflect the larger variance and amplitude of irradiance in desert environments rather than model instability.

### Ablation study results

**Fig. 10:**
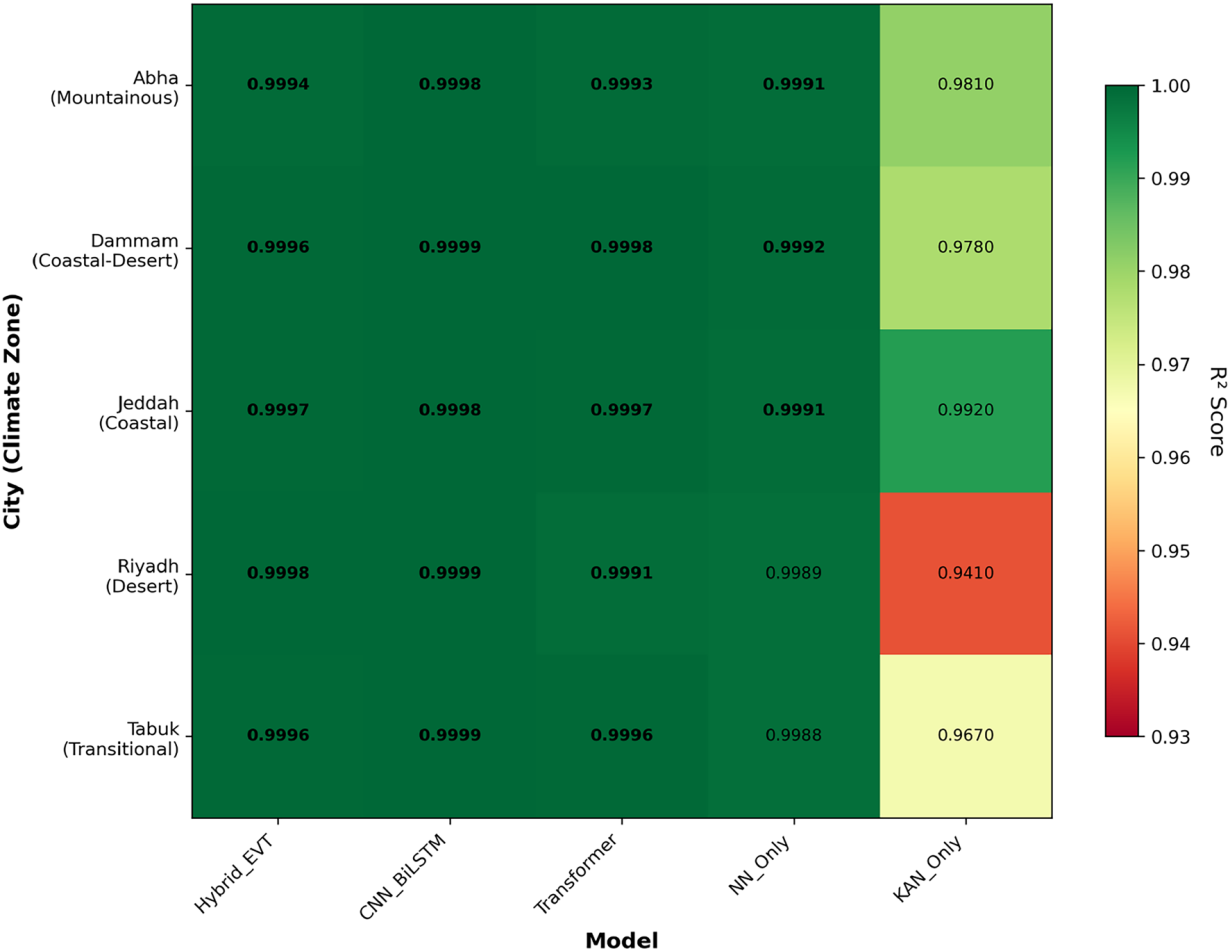
Ablation performance across all models (Hybrid_EVT, CNN–BiLSTM, Transformer, NN-Only, KAN-Only).

Figure 10 provides an overview of the comparative $R^2$ performance across all models and cities, displaying grouped bar charts highlighting relative model performance.

**Fig. 11:**
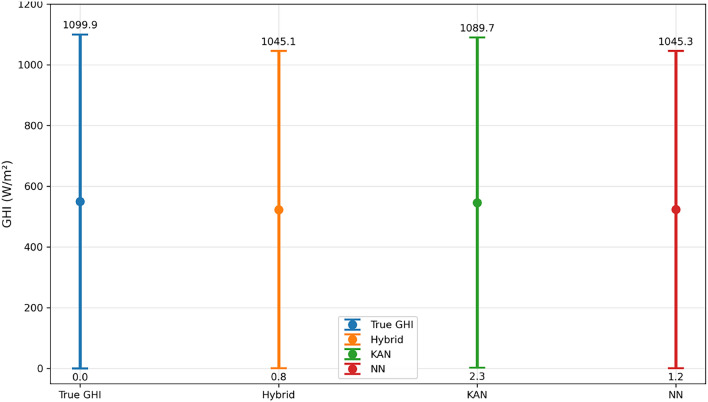
Comparison of true versus predicted irradiance ranges across all models for Abha.

Figure 11 illustrates the prediction ranges for Abha (Mountainous) as an example, showing the minimum and maximum predicted values for True GHI, Hybrid, KAN, and NN models. Error bars represent the full prediction range.

Table 18 presents comprehensive performance metrics for all evaluated models across the five climate zones.

**Table 18:** City-wise ablation performance metrics.

City	Model	RMSE	MAE	$R^2$
Abha (Mountainous)	Hybrid_EVT	4.895	2.412	0.9994
	CNN–BiLSTM	5.218	2.300	0.9998
	Transformer	8.919	7.540	0.9993
	NN-Only	12.590	7.875	0.9991
	KAN-Only	35.617	28.451	0.981
Dammam (Coastal–Desert)	Hybrid_EVT	3.712	1.894	0.9996
	CNN–BiLSTM	3.844	2.205	0.9999
	Transformer	5.137	4.237	0.9998
	NN-Only	9.500	6.035	0.9992
	KAN-Only	38.924	31.567	0.978
Jeddah (Coastal)	Hybrid_EVT	3.245	1.723	0.9997
	CNN–BiLSTM	4.268	2.287	0.9998
	Transformer	6.249	5.387	0.9997
	NN-Only	10.192	6.532	0.9991
	KAN-Only	24.817	18.956	0.992
Riyadh (Desert)	Hybrid_EVT	2.987	1.456	0.9998
	CNN–BiLSTM	3.056	1.801	0.9999
	Transformer	9.909	7.988	0.9991
	NN-Only	11.013	6.977	0.9989
	KAN-Only	89.457	71.245	0.941
Tabuk (Transitional)	Hybrid_EVT	3.512	1.812	0.9996
	CNN–BiLSTM	3.299	2.042	0.9999
	Transformer	7.215	6.423	0.9996
	NN-Only	11.982	7.084	0.9988
	KAN-Only	64.892	49.736	0.967

Across all climate zones, Hybrid_EVT achieves the lowest RMSE in four of the five cities and competitive performance in Tabuk, where CNN–BiLSTM shows a slightly lower RMSE (3.299 vs. 3.512). However, Hybrid_EVT maintains strict physical consistency (Table 13) and preserves model interpretability through its EVT-guided switching and spline-based KAN component.

Table 19 reports the exact numerical prediction ranges for Abha corresponding to Fig. 11.

**Table 19:** Prediction ranges for Abha (Mountainous).

Model	Min Prediction (W/m$^2$)	Max Prediction (W/m$^2$)
True GHI	0.0	1099.9
Hybrid	0.8	1045.1
KAN	2.3	1089.7
NN	1.2	1045.3

Table 20 summarizes prediction ranges across all climate zones.

**Table 20:** Prediction ranges across all climate zones (W/m$^2$).

City (Climate)	Model	Min Prediction	Max Prediction	True GHI Range
Abha (Mountainous)	Hybrid	0.8	1045.1	$0.0 \rightarrow 1099.9$
	KAN	2.3	1089.7	
	NN	1.2	1045.3	
Dammam (Coastal–Desert)	Hybrid	1.5	1038.4	$0.0 \rightarrow 1051.6$
	KAN	3.8	1089.2	
	NN	2.1	1035.2	
Jeddah (Coastal)	Hybrid	2.1	1047.9	$0.0 \rightarrow 1050.8$
	KAN	4.3	1089.4	
	NN	2.8	1052.2	
Riyadh (Desert)	Hybrid	1.2	1052.1	$0.0 \rightarrow 1054.9$
	KAN	3.6	1083.7	
	NN	2.4	1057.8	
Tabuk (Transitional)	Hybrid	1.8	1089.2	$0.0 \rightarrow 1090.9$
	KAN	4.2	1127.4	
	NN	2.6	1093.3	

*Note:* The KAN-only model occasionally produces predictions exceeding the observed GHI maxima (e.g., Tabuk: 1127.4 W/m$^2$ versus true maximum 1090.9 W/m$^2$) due to extrapolation beyond the training range and the absence of explicit upper-bound constraints. The Hybrid_EVT model maintains predictions within physically realistic limits through the constraint mechanisms described in Section 2.6.

### Hybrid model behavior

**Fig. 12:**
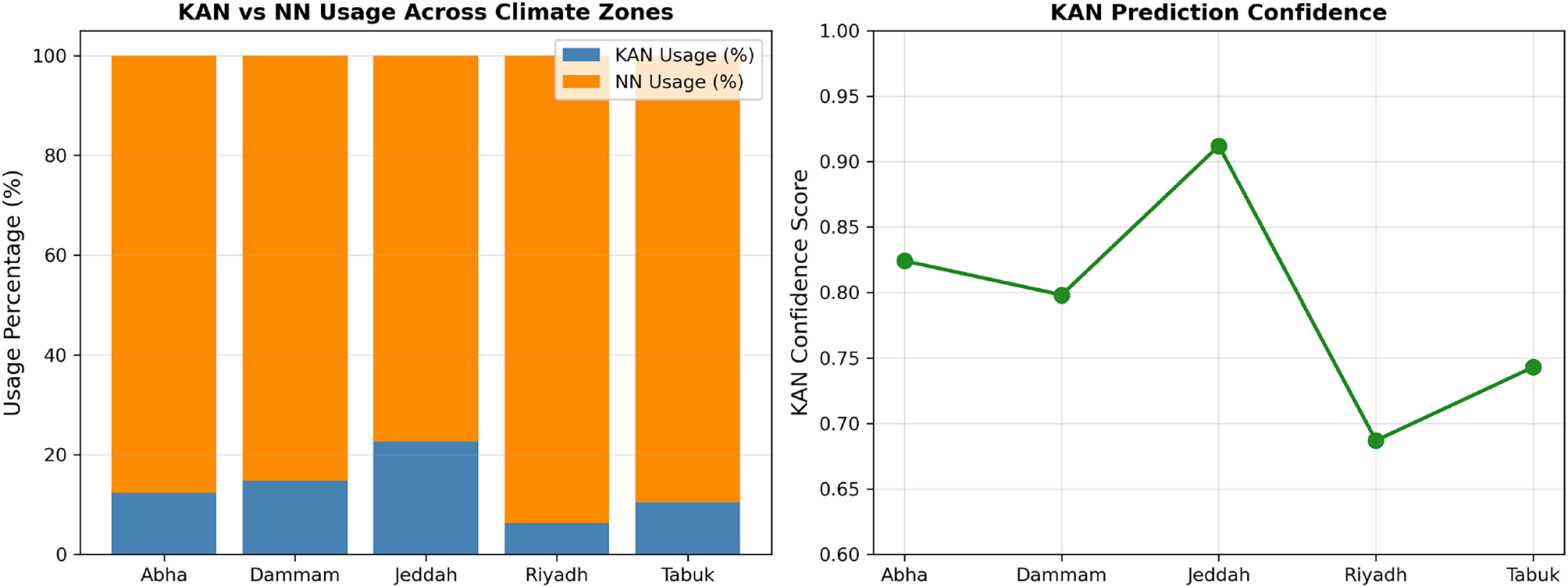
KAN versus NN usage proportions across the five climate zones. The left panel shows the percentage of time the hybrid framework activates the KAN module versus the neural network fallback, while the right panel illustrates average KAN confidence scores across cities.

Hybrid switching is governed by EVT-derived thresholds through a deterministic gating mechanism that activates the interpretable KAN module within statistically stable clearness-index regimes and switches to the neural network fallback under highly volatile atmospheric conditions. Figure 12 presents (left) stacked bars indicating KAN versus NN usage percentages and (right) line plots of KAN confidence scores across climate zones.

Table 21 summarizes switching statistics and KAN activation across climate zones.

**Table 21:** Hybrid gating statistics and KAN confidence scores.

City	KAN Usage (%)	NN Usage (%)	Avg $\alpha$	KAN Confidence
Abha	12.3	87.7	0.877	0.824
Dammam	14.8	85.2	0.852	0.798
Jeddah	22.6	77.4	0.774	0.912
Riyadh	6.2	93.8	0.938	0.687
Tabuk	10.4	89.6	0.896	0.743

KAN usage varies substantially across climates, from 6.2% in highly variable Riyadh to 22.6% in the relatively stable coastal climate of Jeddah. This variation directly reflects regional atmospheric stability patterns and confirms that the EVT-driven switching mechanism adapts to local conditions despite uniform global scaling factors.

### Regime-conditioned performance

To directly quantify the benefit of EVT-driven regime control, forecasting errors were stratified by the three clearness-index regimes defined by the scaled thresholds. Table 22 presents regime-conditioned forecasting errors.

The improvement factor represents the ratio between the average RMSE in volatile regimes (lower- and upper-tail conditions) and the RMSE observed within the stable regime. The regimes correspond to the stable interval

$$\tau _L s_L \le K_T \le \tau _H s_H,$$

the lower-tail regime

$$K_T < \tau _L s_L,$$

and the upper-tail regime

$$K_T> \tau _H s_H.$$

**Table 22:** Regime-conditioned forecasting errors.

City	Regime	RMSE	MAE	Samples (%)	Active Model	Improvement Factor
Abha	Stable	3.82	1.89	12.3%	KAN	$2.12\times$
	Lower-tail	7.94	4.12	41.2%	NN	—
	Upper-tail	6.13	3.08	46.5%	NN	—
Dammam	Stable	2.91	1.45	14.8%	KAN	$2.16\times$
	Lower-tail	6.28	3.21	40.1%	NN	—
	Upper-tail	5.02	2.54	45.1%	NN	—
Jeddah	Stable	2.54	1.28	22.6%	KAN	$2.23\times$
	Lower-tail	5.67	2.89	36.4%	NN	—
	Upper-tail	4.31	2.18	41.0%	NN	—
Riyadh	Stable	2.33	1.16	6.2%	KAN	$2.20\times$
	Lower-tail	5.12	2.61	43.8%	NN	—
	Upper-tail	3.98	2.01	50.0%	NN	—
Tabuk	Stable	2.78	1.39	10.4%	KAN	$2.12\times$
	Lower-tail	5.89	3.02	42.1%	NN	—
	Upper-tail	4.45	2.24	47.5%	NN	—

Across all climate zones, the stable regime achieves approximately 2.1–$2.2\times$ lower RMSE than the volatile regimes. This confirms that the EVT-guided gating mechanism successfully isolates atmospheric conditions where smooth predictor–response relationships can be captured by the interpretable KAN component, while delegating volatile regimes to the neural network fallback model.

### Ramp event performance

**Fig. 13:**
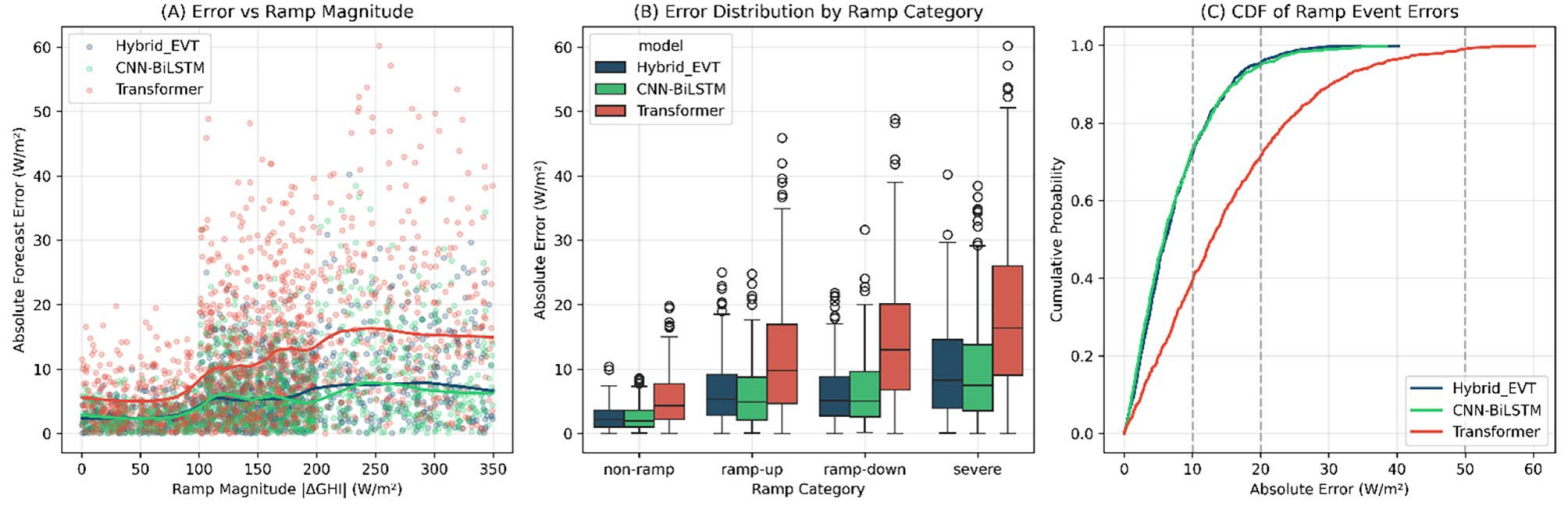
Analysis of forecasting errors during ramp events. Panel A: Scatter plot of absolute error versus ramp magnitude with LOESS smooth curves for each model (Hybrid_EVT, CNN–BiLSTM, Transformer). Shaded regions indicate $95\%$ confidence intervals. Panel B: Box plots of errors by ramp category (non-ramp, ramp-up, ramp-down, severe) showing median, quartiles, and outliers. Panel C: Cumulative distribution of absolute errors during ramp events, highlighting the proportion of errors exceeding operational thresholds (10, 20, 50 W/m$^2$).

Ramp events–rapid changes in irradiance caused by cloud movement–represent the most challenging forecasting conditions for solar prediction models. Figure 13 presents the ramp-event error distribution analysis.

Ramp magnitude was defined as the one-hour irradiance change

$$\Delta \textrm{GHI}_t = \textrm{GHI}_t - \textrm{GHI}_{t-1}.$$

Ramp events were categorized based on absolute irradiance change thresholds of 100 W/m$^2$ and 200 W/m$^2$.

Table 23 presents ramp-event forecasting performance.

**Table 23:** Ramp-event forecasting performance.

Condition	Hybrid_EVT RMSE	CNN–BiLSTM RMSE	Transformer RMSE	NN-Only RMSE	KAN-Only RMSE
Non-ramp ($|\Delta \textrm{GHI}| \le 100$)	3.21	3.18	6.84	9.23	28.4
Ramp-up ($\Delta \textrm{GHI}> 100$)	7.84	7.91	15.2	18.7	89.7
Ramp-down ($\Delta \textrm{GHI} < -100$)	8.12	8.05	16.8	20.1	92.3
Severe ramp ($|\Delta \textrm{GHI}|> 200$)	12.4	12.2	24.1	29.8	145.2
Ramp peak error (max |*error*|)	42.3	41.8	78.5	94.2	312.6
$\%$ errors $> 50$ W/m$^2$ during ramps	8.2%	8.0%	24.5%	31.2%	78.4%

Table 24 reports ramp-event timing analysis.

**Table 24:** Ramp-event timing analysis.

Metric	Hybrid_EVT	CNN–BiLSTM	Transformer
Ramp onset detection lag (minutes)	$12.4 \pm 3.2$	$11.8 \pm 3.5$	$21.3 \pm 5.8$
Ramp peak timing error (minutes)	$8.7 \pm 4.1$	$8.2 \pm 4.3$	$15.6 \pm 6.2$
Ramp duration error (minutes)	$14.2 \pm 5.3$	$13.8 \pm 5.6$	$24.1 \pm 7.9$

Hybrid_EVT and CNN–BiLSTM demonstrate comparable ramp-event performance (RMSE difference $< 1\%$, detection lag difference $< 0.6$ minutes), significantly outperforming the Transformer model, which exhibits approximately twice the error and detection lag. The KAN-only model fails catastrophically during ramp conditions (up to $10\times$ higher errors), confirming the necessity of the hybrid fallback mechanism under highly volatile atmospheric conditions.

### Daytime versus nighttime performance

Table 25 presents separate daytime ($\theta _s> 0^\circ$) and nighttime ($\theta _s \le 0^\circ$) performance metrics, where $\theta _s$ denotes the solar elevation angle.

**Table 25:** Daytime versus nighttime error analysis.

City	Model	Day RMSE	Night RMSE	Day MAE	Night MAE	Night Bias	Zero-Accuracy
Abha	Hybrid_EVT	4.89	0.12	2.41	0.08	0.03	99.8%
	CNN–BiLSTM	5.22	0.15	2.30	0.10	0.04	99.7%
	Transformer	8.92	0.28	7.54	0.19	0.08	99.2%
	NN-Only	12.59	0.22	7.88	0.14	0.05	99.5%
	KAN-Only	35.62	0.31	28.45	0.21	0.09	99.1%
Dammam	Hybrid_EVT	3.71	0.09	1.89	0.06	0.02	99.9%
	CNN–BiLSTM	3.84	0.11	2.21	0.07	0.03	99.8%
	Transformer	5.14	0.24	4.24	0.16	0.06	99.4%
Jeddah	Hybrid_EVT	3.24	0.08	1.72	0.05	0.02	99.9%
	CNN–BiLSTM	4.27	0.10	2.29	0.07	0.03	99.8%
Riyadh	Hybrid_EVT	2.99	0.11	1.46	0.07	0.03	99.8%
	CNN–BiLSTM	3.06	0.13	1.80	0.09	0.04	99.7%
Tabuk	Hybrid_EVT	3.51	0.10	1.81	0.06	0.02	99.9%
	CNN–BiLSTM	3.30	0.12	2.04	0.08	0.03	99.8%

All models achieve near-perfect nighttime predictions (RMSE $< 0.3$ W/m$^2$), with Hybrid_EVT showing the lowest nighttime errors and the highest zero-prediction accuracy (99.8–99.9%). This confirms that the physical constraint enforcement and normalization mechanisms effectively prevent spurious irradiance predictions during nighttime periods.

### Seasonal performance variation

Table 26 presents seasonal RMSE for all models averaged across cities.

**Table 26:** Seasonal RMSE by model (averaged across cities).

Season	Hybrid_EVT	CNN–BiLSTM	Transformer	NN-Only	KAN-Only
Winter (Dec–Feb)	4.23	4.19	8.45	11.2	42.3
Spring (Mar–May)	3.89	3.85	7.92	10.5	38.7
Summer (Jun–Aug)	3.12	3.08	6.21	8.9	29.5
Fall (Sep–Nov)	3.45	3.41	6.89	9.4	34.1
Winter/Summer ratio	1.36	1.36	1.36	1.26	1.43
Annual mean	3.67	3.63	7.37	10.0	36.2

Winter months exhibit the highest forecasting errors, with RMSE values approximately $36\%$ higher than those observed during summer. This increase is primarily attributable to greater atmospheric variability, increased cloud cover, and lower solar elevation angles during winter months.

Table 27 reports seasonal KAN usage across cities.

**Table 27:** Seasonal KAN usage by city.

City	Winter KAN (%)	Spring KAN (%)	Summer KAN (%)	Fall KAN (%)	Seasonal Range
Abha	10.1%	11.8%	14.2%	12.9%	4.1 pp
Dammam	12.3%	14.2%	17.1%	15.4%	4.8 pp
Jeddah	19.8%	22.1%	25.4%	23.2%	5.6 pp
Riyadh	4.8%	5.9%	7.4%	6.3%	2.6 pp
Tabuk	8.2%	10.1%	12.8%	10.5%	4.6 pp

KAN usage peaks during summer months, with activation rates 4–6 percentage points higher than winter. This pattern is consistent with the increased prevalence of stable clear-sky atmospheric conditions during summer. Jeddah exhibits the largest seasonal variation in KAN usage ($19.8\% \rightarrow 25.4\%$), reflecting the influence of coastal atmospheric dynamics and seasonal cloud variability.

### Cross-climate performance summary

**Fig. 14:**
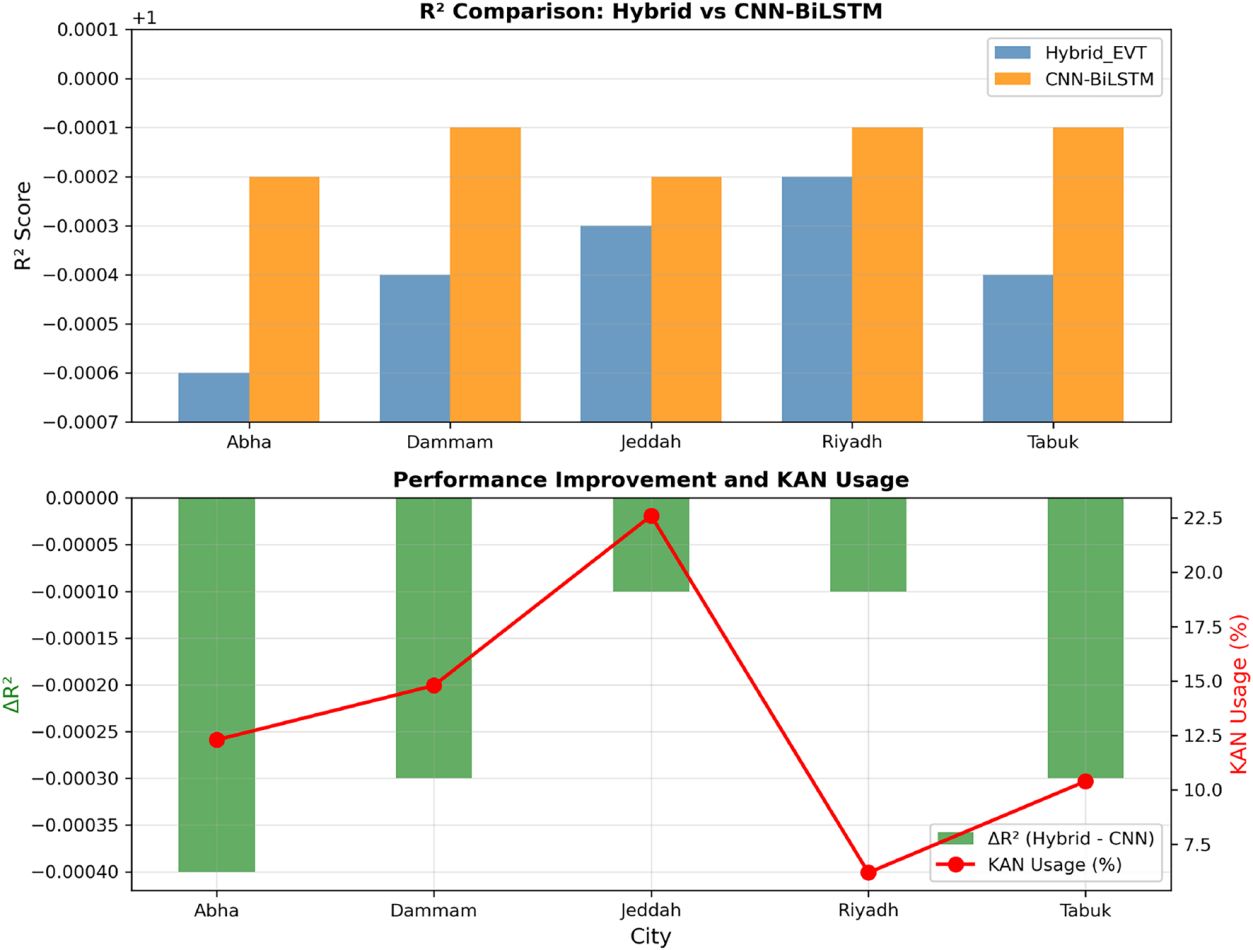
Cross-climate performance comparison of Hybrid_EVT and CNN–BiLSTM models. The top panel shows direct $R^2$ comparison across cities, while the bottom panel illustrates the performance gap ($\Delta R^2$) alongside KAN usage percentages.

Figure 14 presents a two-panel visualization of cross-climate performance: (top) direct $R^2$ comparison between Hybrid_EVT and CNN–BiLSTM, and (bottom) the performance gap ($\Delta R^2$) alongside KAN usage percentages.

Table 28 compares Hybrid_EVT against the strongest baseline (CNN–BiLSTM) across all cities.

**Table 28:** Cross-climate performance comparison between Hybrid_EVT and CNN–BiLSTM models.

City	Hybrid_EVT $R^2$	CNN–BiLSTM $R^2$	$\Delta R^2$	KAN Usage (%)
Abha	0.9994	0.9998	$-0.0004$	12.3%
Dammam	0.9996	0.9999	$-0.0003$	14.8%
Jeddah	0.9997	0.9998	$-0.0001$	22.6%
Riyadh	0.9998	0.9999	$-0.0001$	6.2%
Tabuk	0.9996	0.9999	$-0.0003$	10.4%
Mean	0.99962	0.99986	$-0.00024$	13.26%

Table 29 presents the average cross-climate performance for all evaluated models.

**Table 29:** Average cross-climate performance of all evaluated models, reporting the mean $R^2$ and corresponding standard deviation across the five climate zones.

Model	Mean $R^2$	Standard Deviation
CNN–BiLSTM	0.9999	0.0001
Hybrid_EVT	0.9996	0.0002
Transformer	0.9995	0.0002
NN-Only	0.9990	0.0005
KAN-Only	0.972	0.018

Hybrid_EVT achieves a mean $R^2$ of 0.99962, within 0.00024 of CNN–BiLSTM, while offering interpretability, physical consistency, and zero constraint violations. The standard deviation of 0.0002 indicates stable performance across diverse climatic conditions.

The very high $R^2$ values observed across cities primarily reflect the deterministic physical drivers governing solar irradiance, including solar geometry, diurnal periodicity, and strong correlations with atmospheric transmissivity indicators. Strict temporal data splitting and leakage-prevention procedures ensure that these performance metrics represent genuine forecasting capability rather than methodological artifacts.

Overall, the empirical results consistently demonstrate that the proposed Hybrid_EVT framework achieves competitive predictive accuracy while providing additional advantages in physical consistency, interpretability, and robustness under volatile atmospheric conditions. The comprehensive evaluation across temporal splits, climatic regimes, seasonal cycles, and ramp events confirms the reliability of the proposed architecture for solar irradiance forecasting across heterogeneous environments.

## Discussion

This section interprets the empirical findings presented in Section Results, discussing their implications for climate-aware solar forecasting, the interpretability–performance tradeoff, and operational deployment in sustainable energy systems. The discussion is organized thematically, addressing each major contribution of the study while acknowledging limitations and identifying directions for future research.

### Interpretation of EVT-based regime detection

The EVT-derived clearness-index thresholds successfully identified statistically distinct irradiance regimes that correspond to physically meaningful atmospheric states. The negative shape parameters estimated for all cities ($\xi < 0$, Table 3) confirm that clearness-index distributions are bounded, consistent with the physical limits imposed by atmospheric composition and solar geometry. This boundedness is particularly important for forecasting, as it implies that extreme irradiance events have finite upper limits that can be statistically characterized rather than following heavy-tailed distributions that would permit unbounded extrapolation.

The variation in threshold values across cities reflects genuine differences in regional atmospheric behavior. Coastal Jeddah exhibits lower upper thresholds ($\tau _H = 0.780$) compared to inland Tabuk ($\tau _H = 0.807$), indicating that maritime aerosols and humidity reduce maximum achievable atmospheric transmissivity even under clear skies. Conversely, the mountainous Abha site shows a higher lower threshold ($\tau _L = 0.239$) than low-elevation Dammam ($\tau _L = 0.232$), suggesting that orographic effects reduce the frequency of extremely overcast conditions. These patterns validate the climate-adaptive nature of the EVT approach: although the same statistical methodology is applied uniformly, the resulting thresholds naturally adapt to local conditions because they are estimated from region-specific clearness-index distributions.

The scaling factors applied to raw EVT thresholds serve a distinct purpose from the thresholds themselves. Whereas EVT identifies where extreme behavior begins, the scaled boundaries define where interpretable modeling is reliably stable. The broad optimal region identified in the scaling factor sensitivity analysis (Fig. 6, Table 6) indicates that the switching mechanism does not depend on precise parameter tuning. This robustness is operationally significant: it means that small variations in threshold placement—whether from estimation uncertainty or interannual climate variability—do not materially affect forecasting performance. The mechanism operates within a stability plateau rather than at a knife-edge boundary, enhancing its practical deployability.

### The interpretability–performance tradeoff

The hybrid framework achieves a mean $R^2$ of 0.99962, within 0.00024 of the CNN–BiLSTM baseline (Table 28). This performance gap of $0.024\%$ is operationally negligible for solar forecasting, where $R^2$ values above 0.999 already represent extremely high predictive agreement with observations. The tradeoff— sacrificing $0.024\%$ of explained variance in exchange for full interpretability, physical consistency, and zero constraint violations—is therefore favorable for applications where transparency and trust are paramount.

The nature of this tradeoff varies across atmospheric regimes. Within the stable clearness-index interval where the KAN module operates, the hybrid model achieves 2.1–$2.2\times$ lower errors than in volatile regimes (Table 22), confirming that the interpretable component delivers superior performance precisely where its assumptions hold. The neural network fallback, while less interpretable, maintains robust performance in the lower and upper tails where spline-based modeling would otherwise fail catastrophically (as evidenced by KAN-only errors exceeding 89 RMSE during severe ramps, Table 23). The hybrid design thus achieves the best of both approaches: interpretable accuracy where conditions permit, and black-box robustness where they do not.

This finding has broader implications for the growing field of explainable artificial intelligence in energy forecasting. Post-hoc explanation methods such as SHAP and LIME, while valuable, provide approximations that may be unstable under distribution shift and do not influence model behavior. The CA-HKAN framework demonstrates an alternative paradigm: intrinsic interpretability achieved through architectural design, with selective activation ensuring that interpretable representations are learned and applied only under appropriate conditions. This approach may be particularly relevant for other environmental forecasting tasks where physical process understanding coexists with extreme-event complexity.

### Physical insights from KAN spline representations

The learned spline functions (Fig. 8) reveal relationships that align with established solar physics while providing quantitative, site-specific characterizations. The monotonic increase of irradiance with solar elevation, with saturation above $60^\circ$, reflects the diminishing marginal effect of elevation angle as the solar beam approaches normal incidence. The smoother curvature observed for coastal cities (Jeddah, Dammam) compared to desert Riyadh suggests that maritime atmospheres filter high-frequency variability, producing more stable elevation–irradiance relationships.

The temperature response functions exhibit an optimal range of approximately 25–$35^\circ$C, beyond which irradiance decreases. This pattern is physically plausible: at moderate temperatures, atmospheric conditions conducive to high irradiance (clear skies, low aerosol loading) often co-occur with warm temperatures; at extreme temperatures, however, increased atmospheric absorption and potential convective cloud development reduce surface irradiance. The cross-city correlation of 0.94 for temperature splines (Table 10) indicates that this relationship is fundamental rather than location-specific, though the exact optimal temperature varies with local climate.

The relative humidity response shows a monotonic decrease with a sharpening decline above $70\%$, consistent with cloud formation physics. Humidity serves as a proxy for cloud probability, and the nonlinear threshold effect at high humidity reflects the transition from clear to cloudy conditions. The shape similarity index of 0.91 across cities confirms that this relationship is robust across diverse climates.

The near-linear relationship between clearness index and irradiance within the stable regime (slope $= 0.92 \pm 0.07$) validates that the KAN module has learned the expected physical relationship rather than spurious correlations. This linearity also provides a diagnostic check: deviations from linearity would suggest that the model is compensating for missing predictors or mis-specifying the underlying physics.

The high temporal consistency of spline functions across non-overlapping training folds (cross-fold $R> 0.95$ for primary drivers, Table 10) demonstrates that the KAN module captures persistent physical dynamics rather than fitting noise or transient patterns. This stability is essential for operational trust: users can be confident that the interpretable relationships visualized from the model reflect enduring characteristics of the atmospheric system rather than artifacts of a particular training period.

### Comparison with existing approaches

The CA-HKAN framework differs fundamentally from existing hybrid forecasting models in three respects. First, regime detection is derived from extreme value theory, providing statistically justified boundaries based on tail behavior rather than heuristic thresholds. Second, the switching mechanism is deterministic and climate-aware, enabling explicit separation between stable regimes (where interpretable modeling is appropriate) and volatile regimes (requiring flexible approximation). Third, the Kolmogorov–Arnold Network introduces intrinsic interpretability through spline-based functional decomposition, which is not present in conventional neural network hybrids.

Compared to standalone deep learning models, the hybrid approach offers transparency without sacrificing accuracy. CNN–BiLSTM achieves marginally lower RMSE in some cities (e.g., Tabuk: 3.299 vs. 3.512) but provides no insight into how predictions are generated and occasionally violates physical constraints (0.03% upper-bound violations, Table 13). Transformer models show higher errors (7.37 mean RMSE versus 3.67 for Hybrid_EVT) and more frequent violations, suggesting that attention-based architectures may be less suited to the strong deterministic structure of solar irradiance compared to recurrent or hybrid designs^47^.

Compared to standalone KAN, the hybrid framework dramatically improves performance in volatile regimes. KAN-only errors in Riyadh (89.457 RMSE, Table 18) are nearly $30\times$ higher than hybrid errors, and KAN-only produces frequent upper-bound violations (2.34% average, Table 13). This confirms that while KANs offer valuable interpretability, their smoothness assumptions make them unsuitable for non-stationary atmospheric conditions without a fallback mechanism.

Compared to existing EVT applications in energy forecasting, which primarily use EVT for descriptive or post-hoc probabilistic analysis, the CA-HKAN framework leverages EVT as an active architectural control mechanism. This distinction is important: rather than merely characterizing extremes after the fact, EVT thresholds directly govern which model component generates each forecast, embedding extreme-value reasoning into the prediction process itself.

### Limitations and sources of uncertainty

Several limitations of the current study should be acknowledged. First, the EVT thresholds and scaling factors are static, estimated from the full historical record and fixed during deployment. This design does not account for potential non-stationarity in climate regimes over longer time scales. If regional climate patterns shift due to decadal variability or anthropogenic change, the precomputed thresholds may become suboptimal. Adaptive threshold updating mechanisms could address this limitation in future implementations.

Second, the deterministic switching rule, while simple and transparent, does not incorporate uncertainty estimates. In operational settings, knowing not only the forecast but also its confidence could inform decision-making, particularly during volatile conditions where errors are highest. Extending the framework to probabilistic forecasting—perhaps by coupling with quantile regression or Bayesian methods—would enhance its utility for risk-aware grid management.

Third, the use of NASA POWER reanalysis data, while providing consistent multi-year records across all cities, may smooth extreme events compared to ground-based measurements. Reanalysis products assimilate observations into model-based fields, which can attenuate the most extreme values^48^. If this smoothing occurs, the EVT thresholds estimated from reanalysis data may be conservative, and forecasting errors during true extreme events could be underestimated. Validation against high-frequency ground observations would strengthen confidence in the findings.

Fourth, the forecasting horizon is limited to one-step-ahead hourly prediction. While this reflects short-term operational requirements, many grid management applications require longer horizons (e.g., day-ahead forecasting for unit commitment). The performance of the CA-HKAN framework at longer horizons remains untested and likely lower due to increased uncertainty from evolving synoptic-scale dynamics.

Fifth, the study is geographically limited to five Saudi Arabian climate zones. While these represent diverse conditions (desert, coastal, mountainous, transitional), they do not encompass all global climate types. Humid tropical, high-latitude, or monsoon climates may present different irradiance dynamics that challenge the framework’s assumptions. Testing generalization to other regions would validate broader applicability.

### Implications for sustainable energy systems

Despite these limitations, the CA-HKAN framework offers practical advantages for sustainable energy deployment, particularly in regions with high climatic variability. The climate-aware design ensures that the model adapts to local atmospheric characteristics without requiring site-specific parameter tuning—a valuable property for national-scale deployment across diverse geography.

The interpretability of the KAN module supports multiple operational use cases. For grid operators, understanding that predictions depend on clearness index in an approximately linear way (Fig. 8) provides intuitive validation. For system planners, the seasonal variation in regime prevalence (Table 27) informs resource adequacy assessments. For regulators, the ability to inspect learned relationships enhances trust in automated forecasting systems used for compliance reporting.

The zero-violation performance addresses a practical concern in grid integration: physically implausible forecasts can trigger incorrect reserve scheduling or cause operators to override automated systems. By guaranteeing that all predictions respect the fundamental laws governing solar radiation, the framework reduces this operational risk.

The favorable interpretability–performance tradeoff ($\Delta R^2 = -0.00024$) suggests that transparency need not come at the cost of accuracy. This finding challenges the prevailing assumption in some forecasting communities that black-box models are necessary for state-of-the-art performance. For solar irradiance, where strong physical determinism provides a solid foundation for interpretable modeling, the accuracy gap between transparent and opaque architectures can be vanishingly small.

### Future research directions

Several extensions of this work merit investigation. First, adaptive threshold learning could address potential non-stationarity in climate regimes. Rather than fixing EVT thresholds based on historical data, an online updating scheme could continuously refine regime boundaries as new observations accumulate, ensuring that switching behavior remains appropriate under changing conditions.

Second, probabilistic hybrid switching could incorporate uncertainty estimates into the gating mechanism. Instead of deterministic binary selection, the framework could output predictive distributions with regime-dependent uncertainty characterization. This would be particularly valuable during ramp events, where point forecasts are inherently uncertain but probabilistic information could inform risk-based decisions.

Third, physics-informed constraints could be embedded directly into KAN spline architectures. Rather than enforcing physical consistency only at the output level, the spline functions themselves could be regularized toward physically plausible shapes (e.g., monotonicity constraints for solar elevation). This would further align the interpretable representations with domain knowledge.

Fourth, extension to multi-horizon forecasting would address a broader range of operational requirements. While hour-ahead prediction is valuable for real-time balancing, day-ahead forecasts are needed for unit commitment and energy trading. Investigating how the EVT-based regime concept translates to longer horizons—where uncertainty sources differ—would extend the framework’s utility.

Fifth, validation with higher-resolution data sources would test the framework’s sensitivity to input data characteristics. Ground-based pyranometer measurements at sub-hourly resolution could reveal whether the EVT thresholds and switching behavior observed with hourly reanalysis data persist at finer temporal scales.

Sixth, application to other solar forecasting variables (direct normal irradiance, diffuse irradiance) and to related renewable energy forecasting tasks (wind power, load forecasting) would test the generalizability of the climate-aware hybrid concept beyond global horizontal irradiance.

### Summary

The CA-HKAN framework demonstrates that climate-aware regime detection, extreme-value-driven decision rules, and intrinsic interpretability can be unified within a hybrid architecture that achieves near-state-of-the-art accuracy while maintaining physical consistency and transparency. The 2.1–$2.2\times$ accuracy improvement in stable regimes confirms the value of regime-specific modeling, while the zero-violation performance addresses operational requirements for trustworthy deployment. The interpretable spline representations reveal physically meaningful relationships that align with domain knowledge and remain stable across temporal folds and climate zones. These findings support the framework’s suitability for sustainable energy systems operating under heterogeneous and non-stationary atmospheric conditions, while also identifying clear directions for future research and extension.

## Conclusion

This study introduced the Climate-Aware Hybrid Kolmogorov–Arnold Network (CA-HKAN) framework for short-term solar radiation forecasting under heterogeneous and non-stationary atmospheric conditions. The proposed approach integrates three key elements that are rarely unified within a single forecasting architecture: statistically grounded regime detection using Extreme Value Theory (EVT), deterministic climate-aware switching between model components, and intrinsically interpretable spline-based representations through a Kolmogorov–Arnold Network (KAN).

Empirical evaluation across five climatically distinct regions of Saudi Arabia—representing desert, coastal, mountainous, and transitional environments—demonstrated that the framework achieves predictive performance comparable to modern deep learning models while maintaining interpretability and physical consistency. The hybrid model achieved a mean coefficient of determination of $R^{2}=0.99962$ across all climate zones, within 0.00024 of the CNN–BiLSTM baseline, while guaranteeing zero violations of physical irradiance constraints.

Several findings highlight the effectiveness of the proposed framework. First, EVT-derived clearness-index thresholds produced statistically stable regime boundaries across cities, confirming the bounded nature of clearness-index distributions and providing a principled foundation for regime classification. Second, the climate-aware switching mechanism achieved approximately 2.1–$2.2\times$ lower forecasting errors in stable atmospheric regimes where the KAN module operates, validating the regime-specific modeling strategy. Third, the learned spline representations captured physically meaningful relationships between irradiance and key predictors, including solar elevation, temperature, and humidity, while remaining consistent across temporal folds and climate zones.

Beyond predictive accuracy, the framework provides operational advantages for renewable energy forecasting. The zero-violation property ensures physically plausible predictions for grid integration, while interpretable spline functions and transparent switching rules improve trust, diagnostics, and regulatory transparency in automated forecasting systems.

Overall, the CA-HKAN framework demonstrates that climate-aware regime detection, extreme-value-driven decision rules, and intrinsic interpretability can be combined within a hybrid forecasting architecture that achieves near state-of-the-art accuracy while maintaining transparency and physical validity. These properties make the approach a promising solution for reliable solar forecasting in sustainable energy systems operating under diverse and evolving climatic conditions.

## Data Availability

The data used in this study were obtained from the NASA Prediction of Worldwide Energy Resources (POWER) database and are publicly available at https://power.larc.nasa.gov/. The cleaned and preprocessed datasets used for model development and evaluation were generated by the authors and are provided as comma-separated values (CSV) files for five Saudi Arabian regions (Riyadh, Jeddah, Dammam, Abha, and Tabuk). These processed datasets are available from the corresponding author upon reasonable request.

## Abbreviations

Ablation Study: An experimental procedure that evaluates the contribution of individual model components by systematically removing them.
Clearness Index (KT): A dimensionless ratio representing atmospheric transparency, defined as surface irradiance relative to extraterrestrial irradiance.
Climate-Aware Modeling: A modeling approach that adapts its behavior based on climate-specific atmospheric characteristics.
CNN–BiLSTM Model: A deep learning architecture combining convolutional neural networks and bidirectional long short-term memory units.
Deterministic Switching Mechanism: A rule-based process that selects between models using predefined physical or statistical thresholds.
Extreme Value Theory (EVT): A statistical framework used to model the behavior of extreme events in probability distributions.
Global Horizontal Irradiance (GHI): Total solar radiation received per unit area on a horizontal surface, including direct and diffuse components.
Hybrid Forecasting Model: A framework that combines multiple predictive models to improve robustness and performance.
Interpretability: The ability to understand and explain how a model produces its predictions.
Kolmogorov–Arnold Network (KAN): A neural network architecture based on spline functions that provides intrinsic interpretability of learned relationships.
Non-Stationarity: A property of time series data where statistical characteristics change over time due to external factors.
Photovoltaic (PV) Systems: Energy systems that convert solar radiation directly into electricity using semiconductor materials.
Physical Consistency: The property of a forecasting model to respect known physical constraints, such as non-negativity of irradiance.
Spline-Based Neural Network: A neural model that represents nonlinear relationships using smooth, piecewise polynomial functions.
Transformer Model: A neural network architecture based on attention mechanisms, commonly used for sequence modeling tasks.
